# Prediction of Poly(A) Sites by Poly(A) Read Mapping

**DOI:** 10.1371/journal.pone.0170914

**Published:** 2017-01-30

**Authors:** Thomas Bonfert, Caroline C. Friedel

**Affiliations:** Institute for Informatics, LMU Munich, Munich, Germany; Rutgers New Jersey Medical School, UNITED STATES

## Abstract

RNA-seq reads containing part of the poly(A) tail of transcripts (denoted as poly(A) reads) provide the most direct evidence for the position of poly(A) sites in the genome. However, due to reduced coverage of poly(A) tails by reads, poly(A) reads are not routinely identified during RNA-seq mapping. Nevertheless, recent studies for several herpesviruses successfully employed mapping of poly(A) reads to identify herpesvirus poly(A) sites using different strategies and customized programs. To more easily allow such analyses without requiring additional programs, we integrated poly(A) read mapping and prediction of poly(A) sites into our RNA-seq mapping program ContextMap 2. The implemented approach essentially generalizes previously used poly(A) read mapping approaches and combines them with the context-based approach of ContextMap 2 to take into account information provided by other reads aligned to the same location. Poly(A) read mapping using ContextMap 2 was evaluated on real-life data from the ENCODE project and compared against a competing approach based on transcriptome assembly (KLEAT). This showed high positive predictive value for our approach, evidenced also by the presence of poly(A) signals, and considerably lower runtime than KLEAT. Although sensitivity is low for both methods, we show that this is in part due to a high extent of spurious results in the gold standard set derived from RNA-PET data. Sensitivity improves for poly(A) sites of known transcripts or determined with a more specific poly(A) sequencing protocol and increases with read coverage on transcript ends. Finally, we illustrate the usefulness of the approach in a high read coverage scenario by a re-analysis of published data for herpes simplex virus 1. Thus, with current trends towards increasing sequencing depth and read length, poly(A) read mapping will prove to be increasingly useful and can now be performed automatically during RNA-seq mapping with ContextMap 2.

## Introduction

Gene expression is regulated at several levels, both transcriptionally and post-transcriptionally. An important role for post-transcriptional regulation is played by the 3’ untranslated regions (UTR) of transcripts, which often contain cis-regulatory elements controlling transcript stability, localization and translation, such as AU-rich elements (AREs) and miRNA-binding sites [1]. Shortening of 3’ UTRs resulting from alternative cleavage and polyadenylation has been shown to result in higher protein levels in proliferating cells [2] and over-expression of oncogenes in cancer cells [3]. Alternative polyadenylation has also been found to be tissue-specific in human [4] and *Drosophila melanogaster* [5] and correlated to mouse [6], zebrafish [7], and *D. melanogaster* [8] development. Thus, identification and quantification of poly(A) site usage is of high relevance in deciphering regulation of RNA transcription and processing.

Next-generation sequencing of RNA (RNA-seq) has become the standard technology for transcriptome profiling and has been applied in many studies for identifying expressed genome regions, both coding and non-coding [9–11], differential gene expression [12, 13], alternative splicing [14, 15], and many more. While RNA-seq can be used to identify poly(A) sites by mapping reads containing part of the poly(A) tail (denoted as poly(A) reads in the following) [9], coverage of poly(A) tails by reads has been found to be very poor in previous studies. For instance, RNA-seq analysis of 69 lymphoblastoid cells with a total of 1.2 billion reads by Pickrell et al. recovered only ∼8,000 putative poly(A) sites with >1 poly(A) read [10].

Due to these limitations, a number of alternative experimental techniques for identifying and quantifying poly(A) sites have been developed based on next-generation sequencing, such as PAS-seq [16], PolyA-seq [17], 3’T-fill [18], and several others (reviewed in [19, 20]). These technologies have been successfully used in many studies to map and identify (alternative) poly(A) sites in yeast [18, 21], *Caenorhabditis elegans* [22], human and other mammals [16, 17, 21, 23] among others. Nevertheless, RNA-seq continues to be the most commonly applied approach for transcriptome profiling and is only rarely combined with additional experiments to identify 3’ or 5’ transcript ends. Accordingly, there is a wealth of RNA-seq data available and continues to become available. Despite this abundance of data, poly(A) reads are not standardly identified in RNA-seq analysis pipelines and the information on the poly(A) sites contained within the data is mostly—but not always—ignored.

In particular, mapping of poly(A) reads in RNA-seq data has already been successfully used to identify poly(A) sites in several herpesviruses, including *human cytomegalovirus* (HCMV) [24], *Kaposi’s sarcoma-associated herpesvirus* (KSHV) [25], and *murine gammaherpesvirus 68* (MHV68) [26]. Most recently, we applied this approach to quantify alternative poly(A) site usage during lytic *human herpesvirus 1* (HSV-1) infection [27]. In this study, all but one annotated poly(A) site were recovered as well as three additional ones. Our results argued against a dominant role of alternative poly(A) site usage during HSV-1 lytic infection that had been previously reported and showed that in contrast to host transcription termination, HSV-1 transcription termination was not disrupted during lytic infection.

The likely reason for the successful recovery of poly(A) sites in these herpesviruses is the relative small genome length and high transcription during lytic infection, resulting in high coverage of transcripts by sequencing reads. Thus, even if read coverage of transcript 3’ ends and particularly poly(A) tails is significantly lower than for other transcript regions, sufficiently high numbers of poly(A) reads can still be recovered. This suggests that with the current trend towards both increasing sequencing depth and read length, mapping of poly(A) reads as a standard routine during RNA-seq mapping has potential merits for identifying poly(A) sites even for species with larger genomes. As a consequence, we extended our context-based RNA-seq mapping approach ContextMap 2 [28] to also map reads containing part of the poly(A) tail and identify corresponding poly(A) sites. Our approach is essentially a generalization of approaches previously used for identifying reads containing part of the poly(A) tail, with the main exception that context information, i.e. information provided by other reads, is also taken into account. This allows automatically and efficiently performing poly(A) read mapping as part of the RNA-seq mapping process without requiring additional programs.

Performance of poly(A) read mapping and identification of poly(A) sites was evaluated on RNA-seq data from the ENCODE project [29] for three cell lines. Predicted poly(A) sites were evaluated against a gold standard set obtained from RNA-PET data, which allows identification of transcript 5’ and 3’ ends [30]. Our mapping-based approach is furthermore compared against an alternative approach based on transcriptome assembly presented recently (KLEAT [31]). With default parameters, poly(A) read mapping has a significantly higher positive predictive value (PPV), i.e. a higher fraction of correct predictions, than the assembly-based approach KLEAT, at the cost of lower sensitivity. With alternative parameters, approximately the same sensitivity (and same PPV) can be obtained as for KLEAT. Here, the advantage of our mapping-based approach is the ∼3-fold lower runtime compared to KLEAT.

While PPV was generally high, sensitivity on all “gold standard” poly(A) sites obtained from RNA-PET was poor for both methods, but improved considerably for poly(A) sites near annotated transcript 3’ ends and increased with transcript read coverage. Combined with the observation that the frequency of known poly(A) signal sequences within 50 nt upstream of RNA-PET poly(A) sites was both lower than previously reported [32] and observed for our predictions, this suggests that a substantial fraction of the “gold standard” poly(A) sites are actually incorrect and sensitivity of poly(A) read mapping is underestimated. Indeed, sensitivity more than tripled if identified poly(A) sites were evaluated on more specific poly(A) sequencing data available for one of the evaluated cell lines [33].

In summary, these results show that poly(A) read mapping can successfully recover poly(A) sites with high precision, in particular if read coverage on the corresponding transcripts is high. While major isoforms of highly expressed genes will always be recovered more confidently, more and more poly(A) sites of lowly expressed and minor isoforms will be detected with increasing sequencing depth. This is further illustrated by a re-analysis of the HSV-1 data where both high PPV and sensitivity are achieved, highlighting the value of poly(A) read mapping for host-pathogen transcriptomics. Thus, by integrating poly(A) read mapping into ContextMap 2, which already supports parallel mapping against both host and pathogen genomes, we additionally extended its suitability for these applications. Moreover, since poly(A) read mapping can now be performed conveniently as part of standard read mapping, without requiring additional software, we expect it to be more commonly applied.

## Materials and Methods

### Integration of poly(A) read mapping into ContextMap 2

Recently, we introduced ContextMap 2, a context-based RNA-seq mapping approach [28], which uses the information provided by other reads aligned to the same general genomic location (the so-called context) to select the best alignment for each read. In the original version of ContextMap 2, reads containing parts of a poly(A) tail were not mapped, thus, we implemented a separate procedure for identifying poly(A) sites in HSV-1 [27]. Poly(A) read mapping and 3’ cleavage site prediction has now been integrated into ContextMap 2, making fully use of the context-based approach (version 2.7.8 or higher, available at https://www.bio.ifi.lmu.de/software/contextmap). This allows automatic and effortless poly(A) site prediction during standard read mapping without the need to implement or install additional procedures.

In brief, both the published and current version of ContextMap 2 consist of five steps. In step 1, a set of initial alignments are determined, which are used to determine contexts in step 2. Essentially, contexts are regions of the genome that are covered by alignments and are separated from other contexts by regions without alignments. Initial alignments are determined with the help of an external short read program. While ContextMap 2 also supports the use of Bowtie [34] and Bowtie 2 [35] (and other programs can be included using the plug-in structure of ContextMap 2), best performance was observed in combination with BWA [36]. Thus, all analyses presented in this article were obtained with BWA as short read alignment program for ContextMap 2.

Alignments determined in step 1 include full (unspliced) alignments, single-split alignments (containing only one splice junction), candidate multi-split alignments (potentially containing > 1 splice junction) as well as partial alignments. Partial alignments are reads where only the beginning or end of the read can be aligned and candidate multi-split alignments consist of several single-split alignments for fragments of the read. These initial alignments are extended in step 3 to obtain multiple alternative alignments for each read. Here, alternative split alignments are generated for full or partial alignments that overlap identified splice junctions. Furthermore, for split alignments alternative positions of the splice site are included. Finally, a unique alignment for each read is determined first for each context in step 4 and then across all contexts in step 5 using a support score calculated from maximum read coverages in predefined windows around the read alignment. Complete multi-split alignments are determined in step 4.

**Fig 1:**
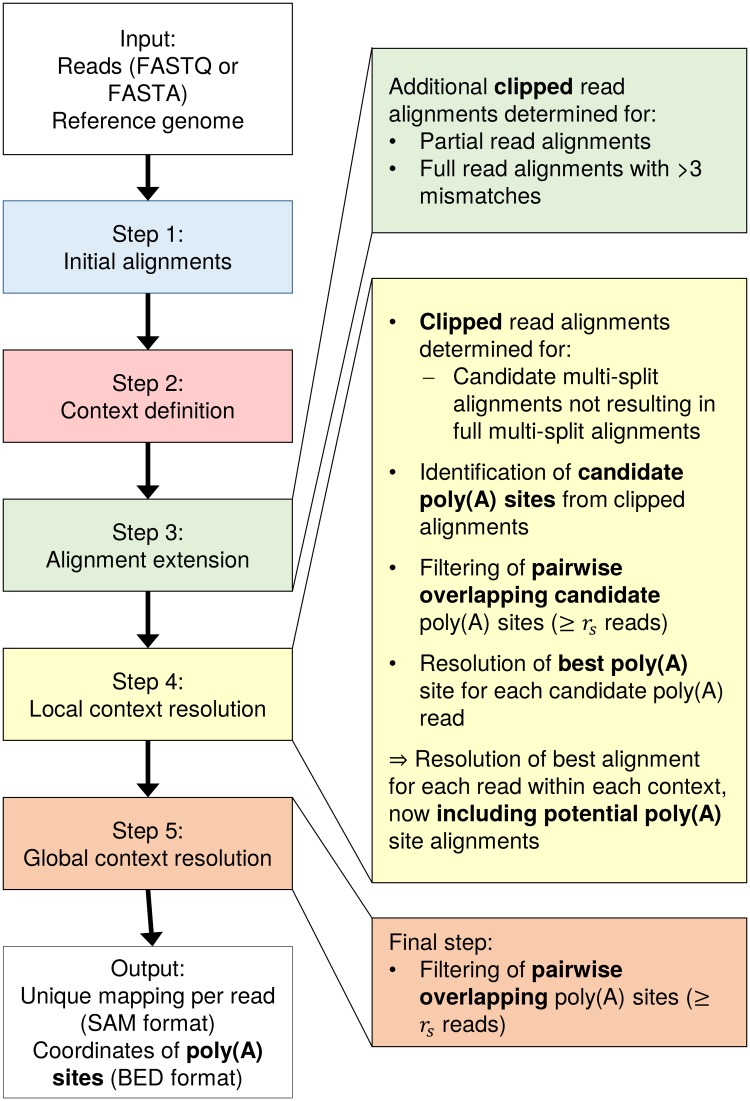
Workflow of poly(A) site identification in ContextMap 2. On the left hand side, the sequence of the five ContextMap 2 steps is indicated. The right hand side illustrates the changes in each step that allow the identification of clipped alignments and poly(A) sites.

A major change in the current version of ContextMap 2 is the support of clipped alignments, i.e. alignments where a prefix and/or a suffix of the read sequence is not aligned (for details see below). Previously, such alignments were discarded, whereas now they are included in the final mapping and are used to determine poly(A) sites. The prediction of poly(A) sites in ContextMap 2 is performed as part of step 4 and is described below, following the description of the clipping method. The coordinates of predicted poly(A) sites are provided as a BED file in addition to the mapping provided in SAM format. Fig 1 illustrates which steps of poly(A) site prediction are performed during which steps of ContextMap 2.

#### Clipping

Clipping is performed in the alignment extension step (step 3 of ContextMap 2), resulting in the inclusion of additional clipped alignments to the set of possible alignments evaluated in subsequent steps 4 and 5. Clipped alignments are determined for:

- Partial read alignments that cannot be extended to valid single-split alignments.
- Full read alignments that have more than three mismatches. In this case, clipped alignments are added to the list of possible alignments in addition to the full alignment, but only if clipping improves the alignment score (defined below).

Essentially, the clipped alignment is a local alignment of the read to the part of the genome the read is aligned to originally, allowing neither insertions or deletions. This local alignment can be determined efficiently in time linear in the read length with a simplified version of the Smith-Waterman algorithm that calculates only the diagonal of the dynamic programming matrix. Per default, we use the same match and mismatch scores used by BWA (match score = 1, mismatch penalty = 4). All clipped alignments with maximum score are added to the set of possible alignments for a read.

Furthermore, if candidate multi-split alignments cannot be combined to an alignment of the complete read in step 4, clipped multi-split read alignments are also determined. In this case, an optimal local alignment beginning at the splice junction closest to the start of the read (in case the multi-split alignment does not extend to the start of the read) or the end of the read (in case the multi-split alignment does not extend to the end of the read) is determined as described above. This allows the combination of clipping with an arbitrary number of splice junctions in the read as well as insertions (modeled as negative-length introns) and deletions (modeled as very small introns).

#### Identification of candidate poly(A) sites

As a first step in poly(A) site prediction, we determine for each clipped read alignment whether it might represent a potential poly(A) site. If it does, a stretch of A’s (or T’s depending on strandedness of sequencing) should begin at the first clipped position in the read. This poly(A) or poly(T) stretch may not necessarily continue until the end of the read as sequencing can continue into primer sequences at the end of fragments and sequencing quality of stretches of the same nucleotide deteriorates rapidly. Furthermore, sequencing errors might disrupt the poly(A) or poly(T) stretch. Thus, we apply the following window-based approach, which allows both a small number of errors and does not require the poly(A) or poly(T) stretch to extend to the end of the read.

**Fig 2:**
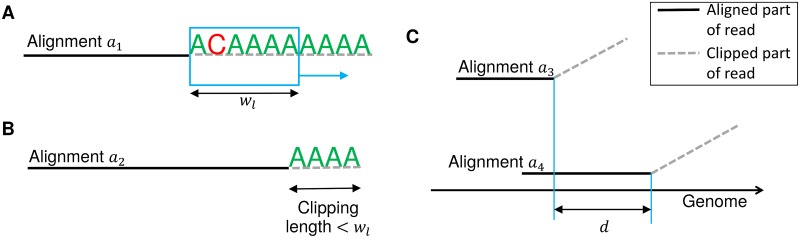
Identification of candidate poly(A) sites. (A) For each alignment, a sliding window of length *w*_*l*_ is shifted along the clipped part of the read sequence and the fraction of A’s (or T’s depending on strandedness of sequencing) is calculated within each window. In this example, the fraction is 5/6 = 0.83 for the first two windows and 6/6 = 1 for all subsequent windows. Thus, at least one window contains ≥ *c*_1_ = 1 A’s and none has < *c*_2_ = 0.7 A’s and this is used as a candidate poly(A) site. (B) In this example, the clipping length is shorter than *w*_*l*_. Accordingly, the window approach cannot be used and all clipped nucleotides are required to be A’s or T’s to predict a candidate poly(A) site, which is the case here. (C) Alignments *a*_3_ and *a*_4_ are considered pairwise overlapping as they are clipped at the same end (dashed lines) and the distance *d* between the start of clipping is smaller than the read length.

A sliding window of length *w*_*l*_ (default *w*_*l*_ = 6 nt) is shifted along the clipped region of the alignment, starting at the first clipped position and ending either at the end of the read or a maximum distance *s*_*l*_ from the clipping start (default *s*_*l*_ = 30 nt), whichever is reached earlier (Fig 2A). For each window, the fraction of A’s or T’s in the read sequence is calculated (depending on strandedness of sequencing). If this fraction is at least *c*_1_ (default *c*_1_ = 1.0) for at least one window and not below *c*_2_ (default *c*_2_ = 0.7) for any window, the clipping start of the alignment is used as a candidate poly(A) site. In case the clipped region of an alignment is shorter than *w*_*l*_ but at least 5 nt, we require that all clipped nucleotides are A’s or T’s, respectively, to treat it as a candidate poly(A) site (Fig 2B).

This approach is similar to previously used approaches for detecting poly(A) sites, such as the one we applied in our analysis of HSV-1 poly(A) sites [27]. While it is highly sensitive, it also produces as lot of spurious results, thus further filtering of candidate poly(A) sites is necessary to correctly identify actual poly(A) sites. Furthermore, clipping may not be performed precisely at the poly(A) site for each poly(A) read, but vary by a few nucleotides to either side if this improves the alignment score, or alternative clipping positions may yield the same alignment score, resulting in several clipped alignments for individual reads. As a consequence, we extended our context-based approach to identify the best poly(A) site among a number of closely located candidate sites based on the number of both clipped read alignments and full read alignments supporting a poly(A) site. This will be described in the following section. We also include a filtering step to exclude spurious hits supported by few reads beforehand.

For filtering, we first identify regions of pairwise overlapping clipped alignments that likely represent the same actual poly(A) site. Two clipped alignments are overlapping if both are clipped at the same end (and only this end) and the distance between the respective clipping start positions is smaller than the maximum read length (see Fig 2C). Alignments clipped at different ends are not considered overlapping as they can only originate from opposite strands. In this way, we identify sets of pairwise overlapping alignments that are connected by pairwise overlaps and cover a region of the genome at most as long as the maximum read length. In case a set covers a larger region of the genome, it is subdivided.

Only sets of pairwise overlapping clipped alignments (and corresponding candidate poly(A) sites) are retained for further analysis if they contain at least *r*_*s*_ (default *r*_*s*_ = 3) alignments containing a candidate poly(A) site. Clipped alignments not supporting a poly(A) site are not further considered for predicting poly(A) sites, but are included in the final mapping if they yield a better score than alternative alignments of the same read.

#### Resolution of poly(A) sites

**Fig 3:**
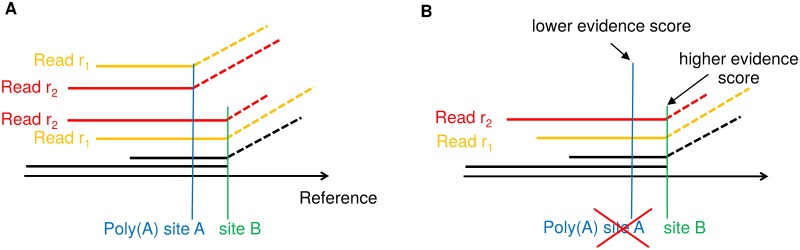
Resolution of poly(A) sites. (A) Example of two overlapping candidate poly(A) sites supported in part (but not only) by alternative alignments of the same reads *r*_1_ and *r*_2_. (B) After calculation of evidence scores, both read *r*_1_ and *r*_2_ are assigned to the poly(A) site with higher evidence (site *B*). Poly(A) site *A* is discarded as it is no longer supported by any reads. Site *B* is supported by ≥ *r*_*s*_ reads with distinct alignments starts and, thus, is included in the final ContextMap 2 output.

Given these sets of pairwise overlapping candidate poly(A) sites, we then identify the best poly(A) site for each read within each set. This means one poly(A) site is chosen for each read among a few closely located ones. For this purpose, ContextMap 2 uses the same evidence score as for the resolution of pairwise overlapping splice sites (part of step 4 of ContextMap 2). The evidence score is calculated from the number of both full and clipped reads supporting the candidate poly(A) site, i.e. the number of reads for which an alignment ends at the poly(A) site (Fig 3). Let *n*_*i*_ be the number of such supporting alignments with *i* mismatches and *m* the maximum number of mismatches allowed. Then the evidence score is defined as:

$$\begin{array}{c} evidence=\sum_{i=0}^{m}\left(w^{i}\cdot n_{i}\right) \end{array}$$ (1)

Here, *w* is a value < 1 (default *w* = 0.3) and is used to decrease the weight of alignments with a larger number of mismatches.

For each read with a candidate poly(A) site within a set of overlapping sites, the clipped alignment to the poly(A) site with highest evidence score is retained and all others are discarded. After this step, there may still be several alternative alignments for a read within the context, including both full alignments of the read and clipped alignments in a different set of overlapping poly(A) sites. These alternative alignments will then be resolved as in the original ContextMap 2 version using a support score that evaluates read alignment density at and around the respective alignments [28]. Thus, at the end of step 4 of ContextMap 2 each read has at most one alignment within each context (full, spliced or clipped). Alternative alignments to different contexts are resolved in step 5 of ContextMap 2 by recalculating support scores and choosing the context for each read with the highest support score for the corresponding alignment. In summary, the main difference to existing poly(A) read mapping approaches is that information provided by other reads is used to resolve alternative clipped alignments for poly(A) reads and identify the correct position of the poly(A) site for each read.

As a last step, after final and unique alignments have been determined for each read, a filtering step is applied again to remove poly(A) sites not sufficiently supported by reads. For this purpose, we define sets of poly(A) sites for which the distance from the first to the last site is at most the read length. If this set of poly(A) sites corresponds to at least *r*_*s*_ identified poly(A) read alignments starting at distinct positions, it is included in the output. Distinct starting positions for alignments are required to eliminate poly(A) sites supported only by PCR duplicates of the same fragment.

### Competing methods

The approach used by ContextMap 2 for prediction of candidate poly(A) sites in individual reads is essentially a generalization of previously used approaches for identifying reads containing part of the poly(A) tail. For instance, Stern-Ginossar et al. classified poly(A) reads for HCMV as reads containing 5 or more consecutive A’s at the 3’ end and at most 1 non-A nucleotide for every 5 A’s [24]. Essentially, this corresponds to the following parameter settings in ContextMap 2: *c*_1_ and *c*_2_ = 5/6 for a window length *w*_*l*_ = 6 and the maximum length of the poly(A) stretch *s*_*l*_ = the maximum read length. Thus, we will not perform a comparison of different strategies for classifying poly(A) reads depending on the existence and length of poly(A) stretches in a read. Different parameter settings of ContextMap 2, which essentially represent such different strategies, will, however, be evaluated on a small training set (see results).

To the best of our knowledge, the only approach for identifying poly(A) sites from RNA-seq data that is not exclusively based on identifying reads containing poly(A) stretches is KLEAT [31]. In contrast to other poly(A) read mapping approaches, it is based on de novo transcriptome assembly as a first step, using established transcriptome assembly approaches, such as Trans-ABySS [37]. In a second step, reads are re-aligned to the resulting contigs and contigs are aligned to the genome. From these alignments, three types of evidence are collected (tails, bridges, and links), which are then used to predict the actual poly(A) sites. Tails are contig sequences ending in poly(A) stretches and are considered as high-confidence candidates. Here, the genome alignment is used to filter false positive results occurring from poly(A) stretches in the genome. Bridges are poly(A)-containing reads aligned to the end of a contig and are included due to problems of assembly programs to assemble poly(A) tails of contigs. Finally, links are read pairs for which the first read aligns to the contig close to its 3’ end and the second read represents a poly(A) sequence.

### Data sets

#### ENCODE data

Evaluation of poly(A) site prediction was performed using ENCODE RNA-seq and RNA-PET data. RNA-PET allows identification of transcript 5’ and 3’ ends [30] and was used to define a gold standard of presumably “true” poly(A) sites. We used ENCODE data for three cell lines (MCF-7, H1-hESC, A549, see Table 1), which was previously also used to evaluate KLEAT. For both RNA-seq and RNA-PET paired-end sequencing was performed with read lengths of 76 and 36 nt, respectively. For our evaluation, replicate data was not pooled before read mapping (in contrast to the published evaluation of KLEAT), allowing both comparison of results between replicates as well as evaluation with realistic sequencing depth.

**Table 1:** ENCODE data used for evaluation.

	RNA-PET			RNA-seq (poly(A) mRNA)		
Cell line	Experiment ID	Rep.	# read pairs	Experiment ID	Rep.	# read pairs
MCF-7	ENCSR000BDD	1	85,864,506	ENCSR000CPT	1	128,178,110
		2	88,182,002		2	131,814,222
H1-hESC	ENCSR000BCC	1	50,218,723	ENCSR000COU	1	125,395,196
					2	107,101,340
A549	ENCSR000BCY	1	91,935,755	ENCSR000CON	1	95,054,259
		2	89,726,158		2	118,364,635

Overview on ENCODE data used for evaluation of poly(A) site mapping. For H1-hESC, only one RNA-PET replicate was available. Read lengths were 76 and 36 nt for RNA-seq and RNA-PET, respectively.

#### RNA-seq data from the study of Pickrell et al

Pickrell et al. performed RNA-seq of 69 lymphoblastoid cell lines derived from unrelated Nigerian individuals [10]. For each sample, prepared libraries were sequenced twice in two different sequencing centers with read lengths of 35 and 46 nt, respectively. For some samples, multiple libraries were prepared, resulting in a total of 161 sequencing data sets with a median sequencing depth of 8.4 million single-end reads. We downloaded the raw sequencing data from the Short Read Archive (accession SRP001540) and the high-confidence poly(A) site predictions by Pickrell et al. from the Supplementary website for the article (http://eqtl.uchicago.edu/RNA_Seq_data/results/).

#### HSV-1 sequencing data

Poly(A) site prediction for HSV-1 was performed using RNA-seq data of newly transcribed RNA from our recently published study on HSV-1 lytic infection [27]. In this study, newly transcribed RNA was labeled in 1h intervals during the first 8h of lytic HSV-1 infection (uninfected cells and 0-1h, 1-2h, 2-3h, 3-4h, 4-5h, 5-6h, 6-7h and 7-8h post infection) and subjected to paired-end sequencing (100 nt reads). Two independent biological replicates were obtained of the full time-course, resulting in 34-61 million read pairs per sample (Gene Expression Omnibus accession: GSE59717).

### Performance measures

RNA-seq and RNA-PET paired-end sequencing reads were mapped against the hg19 human reference genome with ContextMap 2. Gold standard sets of transcript 3’ ends were then determined from each RNA-PET sample by taking the mapped genome position for the read of the read pair that originates from the transcript 3’ end. Both gold standard poly(A) sites and predicted sites from the RNA-seq samples were then clustered in the following way: starting from the left-most poly(A) site on a chromosome, all other poly(A) sites within 25 nt were collected in the same cluster and the process was repeated with the first poly(A) site not within the 25 nt window. For RNA-PET poly(A) site clusters, a 25 nt window was then centered around the poly(A) sites in this cluster. If this window overlapped with the previous cluster window, its start was set one position to the right of the end of this previous window. Clustering of poly(A) sites was performed as there is some heterogeneity in the cleavage position downstream of individual polyadenylation signals [38]. Finally, only RNA-PET clusters containing at least 3 reads were used for evaluation to exclude spurious hits. Clustering of predicted and RNA-PET poly(A) sites was also performed in the KLEAT evaluation [31], however a window length of 100 nt was used for the RNA-PET data. We chose a smaller window size to avoid clustering truly alternative poly(A) sites.

An RNA-PET poly(A) site cluster is then considered a true positive (TP) if at least one poly(A) site of a predicted poly(A) site cluster falls within its 25 nt window and a false negative (FN) otherwise. A predicted poly(A) site cluster is considered a false positive (FP) if none of the predicted poly(A) sites in this cluster fall in an RNA-PET cluster window. Finally, positive predictive value (PPV) and sensitivity are calculated as:

$$\begin{array}{c} PPV=TP/(FP+TP) \end{array}$$ (2)

$$\begin{array}{c} Sensitivity=TP/(TP+FN) \end{array}$$ (3)

Sensitivity and PPV in poly(A) site detection were also compared against read coverage on the last exon of the corresponding transcript (for details see results). Read coverage of an exon was defined as the number of read pairs (i.e. fragments) mapping to the exon divided by the exon length. The number of read pairs mapping to the last exon were calculated using the *featureCounts* program [39] and exon annotation from Ensembl (version 75) [40] with the following call:

featureCounts -f -s 2 -p -g transcript_id -O -M -a gtf-file-with-annotation -o outfile mapping-file.bam

## Results and Discussion

### Parameter selection

In order to select default parameters for poly(A) read mapping in ContextMap 2, we created a small training set from replicate 1 of the ENCODE MCF-7 RNA-seq and RNA-PET data. For this purpose, both RNA-seq and RNA-PET sequencing data was mapped against the hg19 human reference genome using clipping but without poly(A) site prediction. Here, allowing clipping of reads for the RNA-PET data set makes it possible to also map RNA-PET reads that contain a part of the poly(A) tail. Reads mapped to chromosome 6 were then extracted and used to run ContextMap 2 with different parameter settings for poly(A) site prediction to identify parameter settings with a good trade-off between sensitivity and PPV on the RNA-PET data for chromosome 6. The following parameter settings were evaluated in all combinations:

- Length of the sliding window: *w*_*l*_ ∈ [4: 10]
- Maximum number of bases the sliding window is shifted towards the 3’ end of a read: *s*_*l*_ ∈ {10, 15, 20, 25, 30, 1000}
- Minimum fraction of A’s or T’s for at least one window: *c*_1_ ∈ [0.7: 1.0] in increments of 0.1
- Minimum fraction of A’s or T’s for any window: *c*_2_ ∈ [0.3: 1.0] in increments of 0.1
- Minimum number of poly(A) reads supporting a set of pairwise overlapping poly(A) sites: *r*_*s*_ ∈ [2: 5]

**Fig 4:**
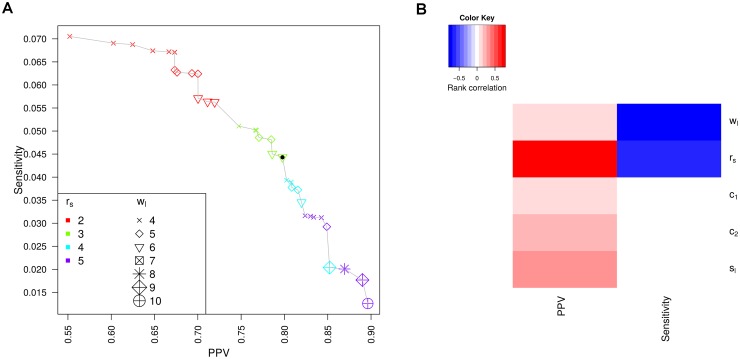
Parameter training results. (A) Comparison of PPV and sensitivity for evaluated parameters. Results are only shown for parameter combinations for which no other combination has a higher PPV at the same or higher sensitivity or a higher sensitivity at the same or higher PPV (i.e. locally “optimal” parameter combinations). Results for the default parameter choice are indicated by a filled black circle. Colors and symbols indicate the values for *r*_*s*_ (minimum number of poly(A) reads required) and *w*_*l*_ (window length), respectively. (B) Heatmap illustrating the (spearman) rank correlation between PPV and sensitivity for each parameter across all evaluated parameter combinations.

Fig 4A illustrates sensitivity and PPV for the evaluated parameters. Here, only results for parameter combinations are shown for which no other parameter combination has a higher PPV at the same or higher sensitivity or a higher sensitivity at the same or higher PPV. The maximum sensitivity reached is relatively low at ∼0.07 and can only be obtained at the cost of a relatively low PPV (0.55). Since we considered this PPV too low for practical applications, we chose the default parameter combinations for ContextMap 2 as those parameter combinations for which the PPV was closest to 0.8. This resulted in a choice of *w*_*l*_ = 6, *s*_*l*_ = 30, *c*_1_ = 1, *c*_2_ = 0.7 and *r*_*s*_ = 3 (marked by the filled black circle in Fig 4A). Sensitivity for the default parameter combination on the training set was ∼0.044.

As expected, the training results show a clear trade-off between sensitivity and PPV, i.e. the higher sensitivity, the lower PPV and vice versa. When correlating values for each of the five parameters against both sensitivity and PPV, we found that sensitivity was strongly negatively correlated to both *w*_*l*_ and *r*_*s*_ (rank correlation -0.73 and -0.63, respectively), but showed little overall correlation to the other three parameters (see Fig 4B). In contrast, PPV was strongly positively correlated to *r*_*s*_ only (rank correlation 0.79), but only weakly to *w*_*l*_ (0.07). Among the remaining parameters, *s*_*l*_ showed the strongest correlation with PPV (0.29). Thus, sensitivity can be increased by reducing the window length or the minimum number of poly(A) reads required for predicting a poly(A) site. In contrast, PPV can be increased by increasing the minimum number of poly(A) reads as well as increasing the length of the read region evaluated for the presence of poly(A) stretches.

These results are also reflected in the locally “optimal” parameter combinations in Fig 4A. Parameter combinations with the same value of *r*_*s*_ essentially cluster together with regard to PPV and sensitivity, with PPV increasing and sensitivity decreasing with increasing values of *r*_*s*_. Furthermore, among each set of parameter combinations with the same value of *r*_*s*_, sensitivity increased with decreasing window length *w*_*l*_ but PPV increased. Despite the low overall correlation of the remaining parameters to sensitivity and PPV, they showed a strong influence on PPV and sensitivity if *w*_*l*_ and *r*_*s*_ were fixed. For our default choice of *w*_*l*_ = 6 and *r*_*s*_ = 3, both *c*_2_ and *s*_*l*_ showed a strong positive correlation to PPV (0.68 and 0.57, respectively) and all three parameters showed some negative correlation to sensitivity (-0.53 for *c*_2_, -0.31 for *c*_1_, and -0.2 for *s*_*l*_). Thus, all parameters are relevant for performance in poly(A) site prediction but window length *w*_*l*_ and the minimum poly(A) read support *r*_*s*_ appear to be most decisive.

### Comparison to KLEAT on ENCODE data

Poly(A) site mapping using ContextMap 2 with default parameters was compared against results obtained with KLEAT version 1.0 using Trans-ABySS for transcript assembly. Transcript assembly was performed with k-mer lengths of 32, 52 and 72 and KLEAT poly(A) site predictions with at least 3 tail and bridge reads were evaluated. We also aimed to evaluate version 2.0 of KLEAT, but the program produced error messages for the selected samples.

Evaluation was performed separately for each replicate of the RNA-seq data against both replicates of the RNA-PET gold standard data (Table 2). As described in the methods section, both gold standard and predicted poly(A) sites within 25 nt were clustered before evaluation. These results showed that ContextMap 2 always outperformed KLEAT with regard to PPV. This was particularly pronounced for the H1-hESC data set, where ContextMap 2 obtained a PPV of ∼0.76, whereas PPV of KLEAT was < 0.46. Consistent with the results observed on the training data, this increased PPV came at the cost of a lower sensitivity. Generally, the higher the gains were in PPV, the higher the loss was in sensitivity. Here, the largest gains in sensitivity for KLEAT compared to ContextMap 2 were obtained on replicate 1 of the A549 data set and on the H1-hESC data sets (increases of up to 60%). However, for the same samples, PPV of ContextMap 2 was between 26 and 96% higher than for KLEAT.

**Table 2:** Evaluation results on ENCODE data.

				RNA-PET rep. 1		RNA-PET rep. 2	
Data set	Method	Rep.	# Preds.	PPV	Sens.	PPV	Sens.
MCF-7							
	ContextMap 2	1	11,114	0.764	0.043	0.802	0.044
	ContextMap 2	2	11,690	0.78	0.047	0.818	0.047
	KLEAT	1	15,671	0.684	0.055	0.711	0.055
	KLEAT	2	17,353	0.654	0.058	0.68	0.058
A549							
	ContextMap 2	1	5,355	0.943	0.032	0.936	0.03
	ContextMap 2	2	12,903	0.896	0.073	0.887	0.069
	KLEAT	1	11,018	0.745	0.052	0.74	0.049
	KLEAT	2	15,478	0.815	0.08	0.806	0.076
H1-hESC							
	ContextMap 2	1	6,734	0.754	0.082	NA	NA
	ContextMap 2	2	5,370	0.774	0.068	NA	NA
	KLEAT	1	15,425	0.453	0.114	NA	NA
	KLEAT	2	14,668	0.395	0.094	NA	NA

PPV and sensitivity for poly(A) site predictions by ContextMap 2 and KLEAT. Both methods were applied to both RNA-seq replicates for each cell line and corresponding results were evaluated using gold standard sets obtained from both RNA-PET replicates for the corresponding cell line (with the exception of H1-hESC, where only one RNA-PET replicate was available).

It should be noted that even though we used ContextMap 2 to map the RNA-PET data before determining the gold standard set, evaluation of poly(A) site prediction was not biased in favor of ContextMap 2 poly(A) site prediction. When we alternatively used the original mapping of the RNA-PET data provided by ENCODE to determine the gold standard set, the same general trend could be observed, i.e. ContextMap 2 had higher PPV whereas KLEAT had higher sensitivity (see S1 Table). For both the MCF-7 and A549 cell line, PPV increased if we used the ContextMap 2 RNA-PET mapping instead of the ENCODE mapping by 3.6 to 7 percentage points but sensitivity decreased by at most 0.4 percentage points. Interestingly, for the H1-hESC cell line the opposite was observed but again gains, in this case in sensitivity, were higher (4.6–7.6 percentage points) than losses, in this case in PPV (at most 3.4 percentage points). Moreover, gains in PPV (for MCF-7 and A549) or sensitivity (for H1-hESC) were higher for KLEAT than for ContextMap2 if we used the gold standard derived from the ContextMap 2 RNA-PET mapping instead of the original ENCODE mapping. Thus, if anything, using ContextMap 2 for mapping the RNA-PET data improved the results in favor of KLEAT, not ContextMap 2. Accordingly, we chose to use the gold standard based on the ContextMap 2 RNA-PET mapping for any further analysis.

Analysis of the reproducibility of evaluation results between replicates showed that the use of different RNA-PET replicates to derive the gold standard had little influence on the results. Furthermore, performance for different replicates of the RNA-seq data also tended to be similar, with the notable exception of the A549 cell line. Here, both ContextMap 2 and KLEAT showed significantly lower sensitivity but higher PPV in the first compared to the second replicate. For replicate 1, ContextMap 2 identified only 5,355 poly(A) sites compared to 12,903 in the second replicate. For KLEAT the relative difference was less pronounced but still substantial, with 11,018 poly(A) sites identified in the first replicate compared to 15,478 in the second. As noted above, this increased number of predictions on replicate 1 for KLEAT resulted in a dramatically reduced PPV compared to the ContextMap 2 predictions (0.74 vs. 0.94). This suggests that a large fraction of the poly(A) sites additionally identified by KLEAT are actually false positives. Similar conclusions can be drawn for the H1-hESC data, where KLEAT identifies more than twice as many poly(A) sites as ContextMap 2, but has a much lower PPV than the ContextMap 2 predictions.

Interestingly, results for both replicates of the MCF-7 data were highly similar to the training results even though only a small subset of replicate 1 had been used for training and replicate 2 had not been used at all. We thus examined for which parameter combinations we obtained a similar sensitivity on the training data as KLEAT obtained on MCF-7 replicate 1. This was the case for *w*_*l*_ = 6, *s*_*l*_ = 1000, *r*_*s*_ = 2, *c*_1_ = 1.0 and *c*_2_ = 0.7 and resulted in a PPV of 0.72 on the training data. Running ContextMap 2 with these parameters on the full MCF-7 replicate 1 set resulted in a PPV of 0.7 and a sensitivity of 0.056, i.e. almost identical results to KLEAT. Thus, depending on parameter choice, similar sensitivity can be achieved as with KLEAT at the cost of a similarly reduced PPV, or more precise predictions can be obtained at the cost of a reduced sensitivity.

While PPV of KLEAT can likely also be improved by requiring more supporting reads for a prediction, the main disadvantage of this approach remains the substantial runtime, as it includes both an assembly phase and a subsequent alignment phase (Table 3). Here, total CPU time for poly(A) site prediction using ContextMap 2 was significantly lower than both the time required by Trans-ABySS, which is used to calculate the input contigs for KLEAT, and KLEAT applied to the Trans-ABySS output. Thus, total CPU time for poly(A) site prediction using ContextMap 2 was less than a third of what was required by the full KLEAT workflow.

**Table 3:** Runtime of ContextMap 2 and KLEAT.

Data set	Rep.	Method	CPU time (h)
A549	1	Trans-ABySS	116.63
		KLEAT	196.28
		ContextMap	87.55
A549	2	Trans-ABySS	128.87
		KLEAT	193.73
		ContextMap	111.23
MCF-7	1	Trans-ABySS	164.77
		KLEAT	297.22
		ContextMap	133.75
MCF-7	2	Trans-ABySS	162.47
		KLEAT	259.57
		ContextMap	133.63
H1-hESC	1	Trans-ABySS	167.87
		KLEAT	438.57
		ContextMap	92.93
H1-hESC	2	Trans-ABySS	140.82
		KLEAT	182.87
		ContextMap	99.82

Runtime of Trans-ABySS using k-mer lengths of 32, 52 and 72, KLEAT applied to the Trans-ABySS output, and ContextMap 2 using default parameters was obtained using 8 cores on the same machines and measured using the Unix “time” command. CPU time was calculated as the sum of “usr” and “sys” time.

### Evaluation on known transcript 3’ ends

The previous analysis suggested that sensitivity of poly(A) site prediction based on poly(A) read mapping is very low. One possible explanation for this observation is that many of the “gold standard” poly(A) sites identified using RNA-PET do not actually represent “true” poly(A) sites but rather spurious results. To investigate this possibility, we first restricted the gold standard set to more confident RNA-PET clusters supported by more reads. Indeed, sensitivity was increased by 2-fold or more if we required at least 10 reads for a gold standard RNA-PET cluster. Not surprisingly, PPV consequently decreased since a number of correct poly(A) site were now classified as incorrect. However, the effect was not as dramatic as for sensitivity, suggesting that a significant number of RNA-PET clusters with low read numbers do not represent correct poly(A) sites.

As a second analysis, we investigated how PPV and sensitivity changed if we only evaluated RNA-PET clusters within 25 nt of an annotated transcript 3’ end (transcript annotation taken from Ensembl version 75). Since results using either RNA-PET replicate for evaluation were very similar, this analysis was only performed using RNA-PET replicate 1 for each cell line as reference. For this analysis, a transcript was considered a TP if both an RNA-PET and a predicted poly(A) site were within 25 nt of its annotated 3’ end, a FP if there was a predicted poly(A) site within 25 nt of its end but no RNA-PET poly(A) site, and a FN if there was an RNA-PET poly(A) site but no poly(A) site within 25 nt of its 3’ end. Here, only poly(A) site predictions by ContextMap 2 using default parameters were evaluated and poly(A) sites were not clustered. If a transcript had more than one RNA-PET or predicted poly(A) site within 25 nt of its 3’ end, it was only counted once as TP, FP or FN, respectively.

**Fig 5:**
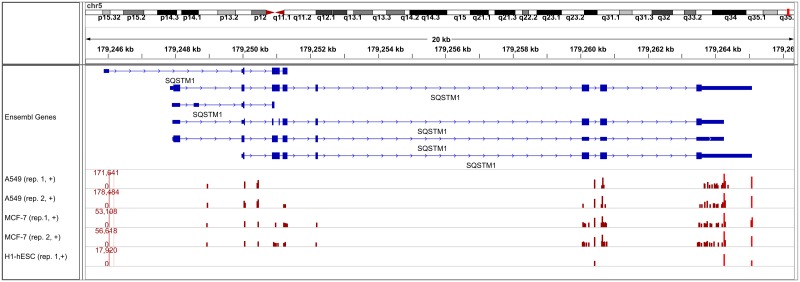
Transcript 3’ ends identified by RNA-PET for an example gene. Poly(A) site clusters identified in the RNA-PET data with at least 20 reads are shown for the *SQSTM1* gene. Only transcripts and clusters on the positive strand are shown. Transcripts annotated in Ensembl are indicated in the top row, with protein-coding exons and untranslated regions indicated by large and small boxes, respectively, introns by lines and strand by arrow heads. Poly(A) site clusters identified in all five RNA-PET samples are shown as boxes in rows 2-6, with the height of the boxes indicating the number of reads for each cluster (in log scale, the range of the y-axis is given in brackets on the left). Light red boxes indicate RNA-PET clusters corresponding to the annotated transcript ends.

Strikingly, the number of transcripts with a predicted poly(A) site within 25 nt of their 3’ end was similar to or only slightly lower than the number of poly(A) site clusters shown in Table 2, whereas the number of transcripts supported by RNA-PET poly(A) sites were only about 15-26% of the total number of RNA-PET clusters. As a consequence, sensitivity increased considerably to 0.19-0.38 (Table 4), but surprisingly PPV increased as well to 0.83-0.96. Thus, these results provide further evidence that a substantial number of RNA-PET clusters are likely measurement errors, in particular if they are not close to a known transcript 3’ end. An example showing that RNA-PET may overestimate the actual number of transcript 3’ ends and accordingly poly(A) sites is given in Fig 5 for the *SQSTM1* gene. While for each sample, two poly(A) site clusters are identified that correspond to annotated transcript ends (marked in lighter red) and also have the highest number of reads, a large number of additional poly(A) sites are suggested by the RNA-PET data. Although they do have lower read counts than the known transcript 3’ ends, they are well above our cutoff of 3 reads (or even 20 reads) and thus were included in the gold standard set for the evaluation on all RNA-PET clusters. Many of those are likely artifacts or represent very rare transcripts. It should be noted here that increasing the cluster size to 100 nt instead of 25 nt would have only little effect on the results in this case, as most of the additional RNA-PET poly(A) site clusters are further away than 100 nt from the two major poly(A) site clusters.

**Table 4:** Evaluation results on known transcripts.

Data set	Rep.	PPV (CM)	PPV (KLEAT)	Sens. (CM)	Sens. (KLEAT)
MCF-7					
	1	0.851	0.836	0.284	0.289
	2	0.894	0.872	0.306	0.298
A549					
	1	0.96	0.932	0.188	0.229
	2	0.919	0.925	0.381	0.353
H1-hESC					
	1	0.826	0.757	0.364	0.386
	2	0.858	0.761	0.31	0.329

PPV and sensitivity were calculated based only on transcripts with either an RNA-PET cluster or predicted poly(A) site within 25 nt of its 3’ end. For each cell line, both RNA-seq samples were analyzed but only replicate 1 of the RNA-PET data was used to derive the gold standard. Results are shown both for the predictions by ContextMap 2 (CM) and KLEAT.

Interestingly, while performance of KLEAT was also generally higher for poly(A) sites close to transcript 3’ ends, the effect on PPV was much more pronounced than for ContextMap 2 (Table 4). As a consequence, although PPV of KLEAT was significantly lower for all predicted poly(A) sites than for ContextMap 2, it was not much lower on the poly(A) sites near transcript ends and in one case even slightly higher. On the other hand, however, relative differences in sensitivity also were reduced, with ContextMap 2 actually outperforming KLEAT in sensitivity in two cases. This observation also suggests that differences in PPV between ContextMap 2 and KLEAT are mostly due to low PPV of KLEAT for poly(A) sites not close to known transcript 3’ ends, i.e. for novel sites.

### Correlation to read coverage

While poly(A) read mapping performs better for poly(A) sites of known transcripts, sensitivity still remains below 40%. We hypothesized that this low sensitivity is mostly due to low sensitivity for transcripts with low expression and thus low read coverage on their 3’ ends. To investigate this possibility, we thus determined read coverage for the last exon of each transcript (read coverage = number of read pairs mapped to the exon divided by exon length, see methods) and correlated this to sensitivity and PPV in identifying the corresponding poly(A) site. For this analysis, we did not normalize to sequencing depth since the actual number of reads is relevant for detecting poly(A) sites not transcript expression as such. At higher sequencing depth, transcripts with lower expression may be detected than at lower sequencing depth due to increased read coverage. Furthermore, expression was calculated for the complete final exon instead of just the region immediately upstream of the poly(A) site as sequencing coverage is generally reduced at transcript ends and expression estimation for small windows tends to be unreliable due to large position-specific variation in sequencing data.

**Fig 6:**
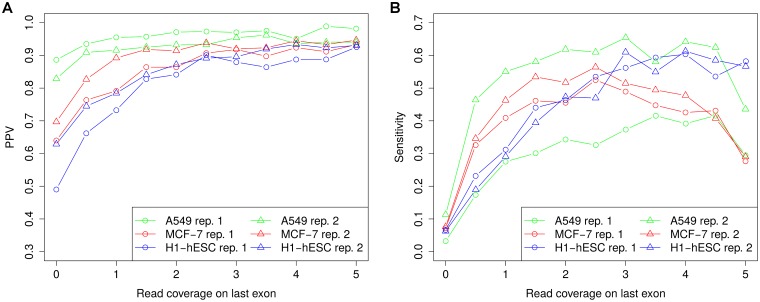
Correlation between read coverage on transcript ends and prediction performance. Transcripts were binned according to the read coverage on the last exon (bin size 0.5) and PPV and sensitivity were calculated separately for each bin. The value on the x-axis indicates the minimum read coverage on the last exon for all transcripts in the corresponding bin and the last bin contains all transcripts with read coverage at least 5.

Fig 6 visualizes both PPV and sensitivity relative to read coverage on the last exon of a transcript for all samples and replicates. This shows that for all RNA-seq samples PPV increased considerably with higher read coverage on transcript ends. Here, highest increases were observed for the MCF-7 and H1-hESC cell lines, for which overall PPV was lower than for the A549 cell line. For the A549 cell line, PPV was already relatively high even for transcripts covered by few reads and did not increase much further. Interestingly, for transcripts with high read coverage on transcript ends, PPV was almost as high in the MCF-7 and H1-hESC cell lines as for the A549 cell line. Furthermore, little differences were observed between replicates. In contrast, sensitivity increased dramatically for all cell lines and showed substantial differences between the two A549 replicates. While for the second replicate, sensitivity reached > 0.6 for transcripts with a read coverage of 2-3 read pairs per nucleotide on the last exon, sensitivity for the first replicate remained below 0.4. Thus, the difference in sensitivity between the replicates cannot be simply explained by the lower sequencing depth of replicate 1 as it is also observed at similar read coverages.

Surprisingly, for the A549 and MCF-7 cell lines, sensitivity did not increase or saturate with even higher read coverage, but rather decreased for read coverages > 4.5 and > 2.5, respectively. We hypothesized that this might be due to increased recovery of minor isoforms of highly expressed genes in the RNA-PET data. If these minor isoforms overlap in the last exon with more highly expressed transcripts of the same gene, read coverage at the 3’ end of these minor isoforms would be overestimated.

**Fig 7:**
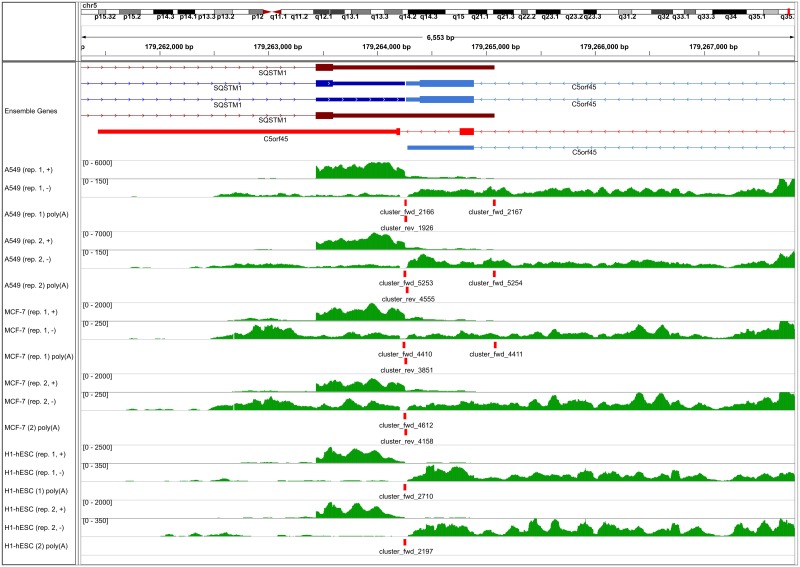
Identified poly(A) sites for example genes. Number of mapped reads for each nucleotide as well as identified poly(A) site clusters are shown for two example genes, i.e. *SQSTM1* and *C5orf45*. Transcripts annotated for both genes in Ensembl are shown in the top row, with protein-coding exons and untranslated regions indicated by large and small boxes, respectively, introns by lines and strand by arrow heads. Transcripts corresponding to the major and minor poly(A) sites (according to the RNA-seq data) are indicated in blue and red (dark: *SQSTM1*, light: *C5orf45*), respectively. For each sample, numbers of mapped reads are shown separately for the two strands (green) and ranges of read numbers are indicated in brackets. Poly(A) site clusters are indicated by red boxes and cluster names indicate the strand: fwd = positive strand, rev = negative strand.

This is exemplified in Fig 7 for gene *SQSTM1*, for which transcripts using two alternative poly(A) sites are annotated, with the downstream poly(A) site used less frequently (see also Fig 5 for RNA-PET clusters for the same gene). Since the last exon of the minor and major isoforms overlap, read coverage on the last exon for the minor isoform appears to be relatively high (∼ 4−17 depending on the replicate). If we calculate read coverage only for the unique part of the last exon of the minor isoform, read coverage is reduced to only 0.26–2.35, with highest coverage observed in the A549 samples. While in this case, this problem could be addressed by evaluating expression in a smaller window upstream of the predicted poly(A) site, this is not a general solution as the minor isoform may also end upstream of the major isoform and thus within an exon of the major isoform. Not surprisingly, the poly(A) site of the minor isoform is only identified in these samples as well as replicate 1 of the MCF-7 cell line, while the more frequently observed poly(A) site is observed in all samples. In contrast, the *C5orf45* gene on the opposite strand shows an example in which transcripts using alternative poly(A) sites do not overlap. Thus, the low read coverage on the last exon for the minor isoform is correctly identified. In this case read coverage is also relatively low on the major isoform (0.44–1.48), explaining why it is not recovered in all samples.

**Fig 8:**
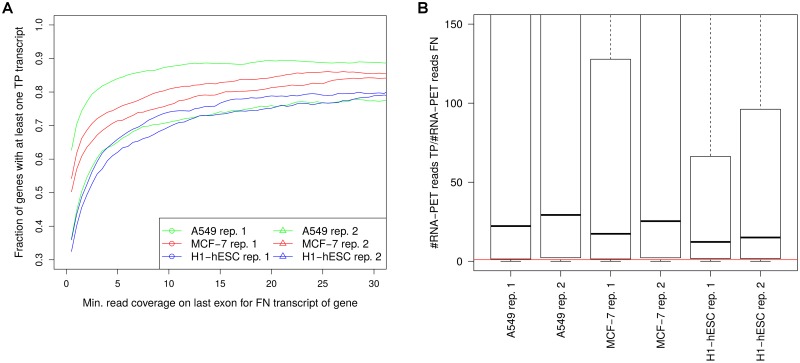
Lower RNA-PET read support for FN transcripts. (A) Fraction of genes with at least one FN transcript 3’ end for which also a TP transcript 3’ end was detected plotted against the read coverage on the last exon of the FN transcript. For this purpose, we identified genes for which at least one FN transcript was observed with read coverage on the last exon at least a value *t*. We then calculated the fraction of these genes with at least a TP transcript and plotted these against increasing values of *t*. (B) Boxplot of the fold-change between the number of reads in the RNA-PET data for the best supported FN and TP transcript for each gene. Here, all genes with at least one FN transcript with read coverage on the last exon ≥ 2 were included. The red horizontal line indicates a fold-change of 1, showing that > 77% of genes had a TP transcript with higher read numbers in the RNA-PET data than the best FN transcript for the same gene.

For a more systematic analysis, we collected all genes with at least one FN transcript (i.e. a transcript with an RNA-PET poly(A) site within 25 nt of its 3’ end but no predicted poly(A) site) and compared the number of RNA-PET reads for FN and TP transcripts of the same gene. For this purpose, we first evaluated for which fraction of genes with at least one FN transcript, at least one FP transcript was also observed (Fig 8A) and correlated this to the read coverage on the last exon of the FN transcript. If more than one FN transcript was observed for the same gene, the one with the highest read coverage on the last exon was chosen. The higher the read coverage was on the last exon for the FN transcript, the higher was the likelihood that a TP transcript was observed for the same gene. In particular, ∼60% of genes with at least one FN transcript with read coverage ≥2 on the last exon also had a TP transcript. For these genes, we then calculated the fold-change in the number of RNA-PET reads between the TP and FN transcript with the highest number of RNA-PET reads among all TP and FN transcripts for this gene, respectively (Fig 8B).

This showed that for the TP transcripts, the number of RNA-PET reads on the 3’ end were on on average 12-29 fold higher than for the FN transcript of the same gene. In particular, for > 77% of genes, the TP transcript had higher read numbers in the RNA-PET data than the best supported corresponding FN transcript, showing that the FN transcripts and corresponding poly(A) sites indeed represent minor isoforms. Thus, despite seemingly high read coverage on the transcript ends, likely due to an overlap with more highly expressed isoforms, their expression is actually low, resulting in few poly(A) reads and accordingly low sensitivity of the poly(A) mapping approach. Alternatively, the relatively low number of reads in the RNA-PET data for these FN poly(A) sites might also be indicative of measurement errors, the frequency of which might also be increased for more highly expressed genes.

### Presence of poly(A) signal sequences

**Fig 9:**
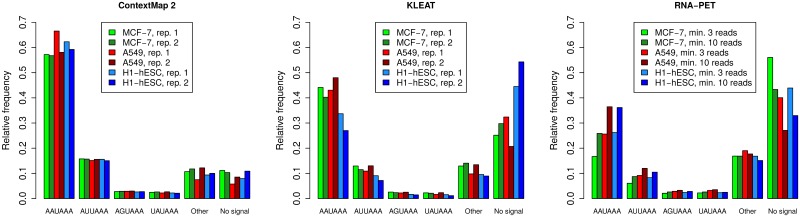
Presence of poly(A) signal sequences. Frequency of poly(A) signal sequences were determined within a 50 nt window upstream of identified poly(A) site clusters. From left to right: predictions of ContextMap 2 (all samples and replicates), predictions of KLEAT (all samples and replicates) and “gold standard” poly(A) site clusters identified from RNA-PET data (all samples, replicate 1) with at least 3 and 10 reads, respectively. Poly(A) signal sequences were determined in the order of overall frequency determined by Beaudoing et al. [32], i.e. first the most frequent AAUAAA signal was searched, then the second-most frequent signal AUUAAA, and so on.

Although the mechanisms leading to the formation of the poly(A) tail and in particular alternative polyadenylation are not yet fully understood, poly(A) signal sequences have been relatively well characterized [32, 41, 42]. The consensus for mammalian poly(A) signals has been described to contain (i) an AAUAAA (or closely related) sequence 15-30 nt upstream of the poly(A) site, (2) a U-rich sequence 0-20 nt upstream of the AAUAAA sequence, and (i) a G/U- or U-rich sequence 0-20 nt downstream of the poly(A) site [42]. While the definition of the U-rich and G/U-rich regions is relatively vague, the presence of the AAUAAA signal or related sequences can be easily verified. To do so, we searched for AAUAAA or related sequences within 50 nt upstream both of the poly(A) sites predicted by ContextMap 2 and KLEAT as well as the “gold standard” poly(A) sites obtained using RNA-PET (see Fig 9 for results on the clustered poly(A) sites and S1 Fig for all poly(A) sites).

These results show that for all predictions and the gold standard, the order with regard to the frequency of poly(A) signal sequences was as previously described by Beaudoing et al. [32], with AAUAAA being most frequent followed by AUUAAA. However, the ContextMap 2 predictions more closely reflected the previously reported frequencies, with average frequency of AAUAAA at 60% (Beaudoing et al.: 58.2%) and of AUUAAA at 15.5% (Beaudoing et al.: 14.9%). In contrast, the same values for the KLEAT predictions were 41.7% and 11.2%, respectively, while the fraction of predictions with no signal at all was at 31.1%, compared to only 9.1% for ContextMap 2. Strikingly, frequencies were even worse for the RNA-PET gold standard, with AAUAAA at 22.8%, AUUAAA at 7.9% and no signal at 46.7%. Although these results improved when including only poly(A) sites with at least 10 reads, i.e. more confident ones, this provides further evidence for high rates of incorrect poly(A) sites in the RNA-PET data. Interestingly, results improved if we analyzed individual RNA-PET poly(A) sites instead of clustered sites, whereas they hardly changed for the predictions (see S1 Fig). This is likely due to the fact that sites with higher support in the RNA-PET data, and therefore higher likelihood to be correct, were selected if we applied the minimum read cutoff to individual sites instead of clusters. In contrast, no such cutoff was applied to the predictions, thus it did not matter much whether we evaluated individual sites or clusters.

In summary, these results confirm our previous observations that poly(A) site predictions of ContextMap 2 with default parameters are more likely correct compared to KLEAT predictions. This may come at the cost of missing poly(A) sites, in particular those that are less frequently used, for instance because they use non-canonical poly(A) signal sequences. It should be noted that neither ContextMap 2 nor KLEAT use the presence of signal sequences for prediction, but it could be used as a post-processing step to further increase PPV. Finally, this analysis provides additional evidence that a significant fraction of RNA-PET poly(A) site clusters do not represent true poly(A) sites and that sensitivity of poly(A) site prediction is likely massively underestimated.

### Evaluation on an alternative gold standard set

As our previous analyses suggested a poor quality of the RNA-PET gold standard, we also performed an evaluation of PPV and sensitivity using data determined with a sequencing protocol specifically developed for poly(A) site analysis. In this case, we used sequencing data for the MCF-7 cell line obtained by the SAPAS (sequencing alternative polyadenylation sites) method by Fu et al. (11, 998, 589 single-end 76 nt reads) [33]. Mapping of this data set with ContextMap 2 and clustering of the obtained poly(A) sites was performed as described for the RNA-PET data. Unfortunately, however, confidence of mapping by ContextMap 2 for the SAPAS data set was very low, i.e. for ∼88% of reads at least two alternative contexts were identified and differences in the scores between the two best contexts was very low (median relative difference = 3%). This means that for the large majority of reads the decision for one of the alternative contexts was made based on very low score differences, likely resulting in a high number of wrong assignments. For comparison purposes, in the RNA-PET data multiple contexts were only identified for 5.9 to 39% of reads and score differences were substantially higher (8.6 to 26.3%), thus allowing more confident mapping. Interestingly, the likely reason why ContextMap 2 mapping confidence is so low on the SAPAS data is actually the higher specificity of this data with regard to poly(A) sites. As illustrated in S2 Fig, RNA-PET reads can be found on all exons of a gene in addition to the transcript 5’ and 3’ ends, while SAPAS reads are limited to the actual poly(A) site. As a consequence, in the SAPAS data, other reads in the same context essentially map to the same locations within the same contexts and thus provide little information for distinguishing alternative mappings for a read in different contexts.

The likely consequence of this low confidence was that PPV on the gold standard calculated from this mapping was very poor (16% for KLEAT and 26% for ContextMap 2, see S2 Table). We thus investigated whether results improved if the SAPAS data were mapped with a different approach. We chose to use BWA as detection of splice junctions was not necessary for this application and BWA also determines clipped read alignments. Thus, the poly(A) site can be determined by a local alignment of the read end even if the read contains an exon-exon junction. Furthermore, most reads should not contain splice junctions anyway as the average length of 3’ UTRs in humans is around 800 nucleotides [43] and reads were only 76 nt long. Based on the resulting BWA mapping, a gold standard was then determined again by clustering the observed poly(A) sites. With the BWA mapping, PPV was again in a similar range as for the RNA-PET data (Table 5).

**Table 5:** Evaluation results on SAPAS MCF-7 data.

Method	Rep.	# Preds.	PPV	Sens.
ContextMap 2	1	11,114	0.802	0.143
ContextMap 2	2	11,690	0.812	0.153
KLEAT	1	15,671	0.625	0.158
KLEAT	2	17,353	0.576	0.161

PPV and sensitivity for poly(A) site predictions by ContextMap 2 and KLEAT for the MCF-7 RNA-seq data using a gold standard set obtained from the SAPAS data on MCF-7 from the study of Fu et al. [33]. Both methods were applied to both RNA-seq replicates for each cell line and corresponding results were evaluated. Mapping of the SAPAS data was performed with BWA.

Consistent with the results on the RNA-PET data, this evaluation showed that ContextMap 2 outperformed KLEAT vastly in PPV, but had lower sensitivity. Notably, however, the difference in PPV was even larger as PPV of ContextMap 2 increased to ∼0.81 (from ∼0.77), whereas PPV of KLEAT decreased to ∼0.6 (from ∼0.69). Sensitivity approximately tripled for both methods, but relative increases were higher for ContextMap 2 (from ∼0.045 to ∼0.15) than for KLEAT (from ∼0.056 to ∼0.16). Thus, relative differences in sensitivity were lower on the SAPAS data than on the RNA-PET data. Accordingly, apart from confirming the general conclusions on the performance differences between ContextMap 2 and KLEAT, this analysis also shows that indeed RNA-PET vastly underestimates sensitivity of poly(A) site prediction from RNA-seq data.

Finally, as mapping with BWA improved results so considerably here, we also repeated mapping for the RNA-PET using BWA (see S3 Table). However, in this case using BWA brought no general improvement. Although for the A549 cell line sensitivity increased, PPV considerably decreased for all cell lines, even more than if we used the original ENCODE mapping. Furthermore, the general conclusions stayed the same, i.e. ContextMap 2 with default parameters outperformed KLEAT with regard to PPV but had lower sensitivity. There are two likely reasons why BWA does not seem to be appropriate for mapping the RNA-PET data. First, read length was quite short at 36 nt and BWA has been shown to perform worse on short reads than on longer ones, in particular also worse than e.g. Bowtie, which was used for the original ENCODE mapping [44]. Second, as illustrated above, the RNA-PET data appears to be more similar to RNA-seq data than to more specific data on poly(A) sites as obtained e.g. by SAPAS and BWA is not a spliced alignment program for use on RNA-seq data. Neither is Bowtie, which may also explain why using a dedicated RNA-seq mapping program such as ContextMap 2 for mapping the RNA-PET data improved results compared to the original ENCODE mapping with Bowtie.

### Re-analysis of the data by Pickrell et al.

The above results showed that high PPV can be achieved when predicting poly(A) sites by mapping poly(A) reads, but that the number of recovered poly(A) sites still remains relatively low. However, compared to the study by Pickrell et al. [10], which found only ∼8,000 poly(A) sites supported by ≥ 2 reads in 161 RNA-seq data sets with 1.2 billion reads in total, results on the ENCODE data still represent a significant improvement (5,000 to 12,000 identified poly(A) sites supported by ≥ 3 reads *per sample* with <130 million read pairs per sample). One crucial factor explaining these improvements is likely the increased read length (76 nt paired-end reads for the ENCODE RNA-seq data compared to 35-46 nt reads for the study by Pickrell et al.), which increases both the number of reads per transcript position and the confidence in the poly(A) read alignments as they contain both more of the transcript sequence encoded in the genome and more of the poly(A) tail.

To investigate whether the original results by Pickrell et al. could be improved by our poly(A) mapping approach, we remapped all 161 data sets from the Pickrell et al. study separately using ContextMap 2. Not surprisingly, default parameters requiring at least 3 reads for each set of overlapping poly(A) sites in each data set resulted only in a small number of poly(A) site predictions (3,852 individual poly(A) sites and 1,425 clusters). Of note, the median number of poly(A) sites identified per sample was as low as 30 and the maximum number observed for any sample only 899. This shows that sequencing depth and read length have a substantial effect on the number of identified poly(A) sites.

For a fairer comparison to the approach applied by Pickrell et al., we also applied ContextMap 2 with parameters better reflecting this approach. Here, the Pickrell et al. method consisted of trimming stretches of at least 4 A’s or T’s from the end of unmapped reads and then mapping the trimmed reads. Subsequently, they discarded matches if the downstream genomic region contained at least 3 A’s or T’s, since one sequencing error at the non-A or T position might then result in an erroneous poly(A) site prediction. Finally, they filtered out potential poly(A) sites supported by < 2 reads (resulting in 7,926 putative sites) and without an AAUAAA motif (the most frequent poly(A) signal sequence, see above) within 50 nt upstream of the poly(A) site (resulting in a final list of 3,481 high-confidence sites).

Accordingly, ContextMap 2 parameters were changed to *r*_*s*_ = 1, *w*_*l*_ = 4, *c*_1_ = 1 and *c*_2_ = 1 (*s*_*l*_ remained at 30), i.e. at least one poly(A) read was required for a set of overlapping sites, at least four A’s or T’s were required and no mismatches were allowed in the stretch of A’s or T’s. Subsequently, the filtering steps used by Pickrell et al. were also applied, resulting in 18,471 putative poly(A) sites and 4,805 high-confidence sites (i.e. supported by at least 2 reads across all data sets and with an AAUAAA motif within 50 nt upstream of the poly(A) site). After clustering, we obtained 3,007 poly(A) site clusters with ContextMap 2 compared to 2,331 clusters for the original high-confidence poly(A) sites identified by Pickrell et al., i.e. an increase of 29%. When comparing the two sets of poly(A) site clusters, we found that ContextMap 2 recovered 81.3% of the poly(A) site clusters identified by Pickrell et al., while only 64.4% of the ContextMap 2 poly(A) site predictions were also identified by Pickrell et al.

To estimate the PPV for the poly(A) predictions unique to either method or common to both, we evaluated which percentage of the identified poly(A) site clusters were within 25 nt of transcript 3’ ends annotated in Ensembl. For poly(A) site clusters identified by both methods, this number was 87%. In contrast, 78% of poly(A) site clusters unique to the ContextMap 2 predictions where within 25 nt of annotated transcript 3’ ends, compared to 74% of poly(A) site clusters unique to the Pickrell et al. predictions. The lower percentage for the unique predictions compared to common ones may be explained by the observation that in both cases poly(A) sites not recovered by the respective other method had significantly lower read counts than poly(A) sites identified by both methods (Wilcoxon rank sum test, p-value < 10^−15^). In summary, these results show ContextMap 2 recovers almost all of the poly(A) sites identified by Pickrell et al. and identifies a larger number of additional sites, for which PPV appears to similar or even better than for poly(A) site predictions identified only by Pickrell et al.

### Replicate reproducibility and influence of sequencing depth

**Fig 10:**
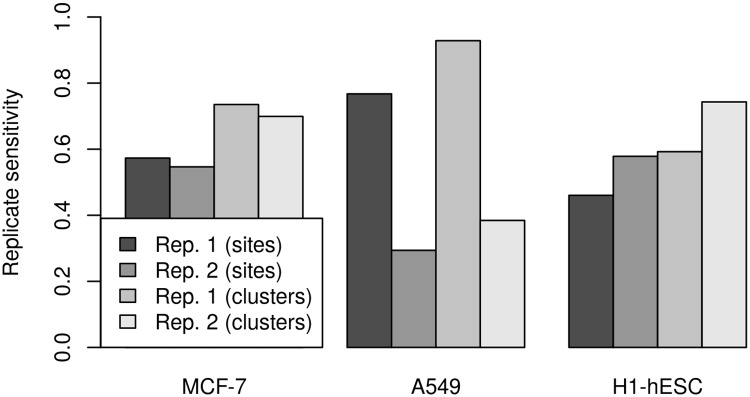
Replicate sensitivity for poly(A) site prediction. For each replicate, the fraction of individual poly(A) site predictions and clusters, respectively, are shown that were also recovered in the other replicate for the same cell line.

As the analysis of PPV and sensitivity for poly(A) site predictions for different replicates of the same cell line indicated considerable variation between replicates, in particular for the A549 cell line, we also performed an analysis of reproducibility between replicates of poly(A) sites predicted by ContextMap 2. When calculating the fraction of either poly(A) sites or poly(A) site clusters (for a cluster size of 25 nt) also identified in the respective other replicate (denoted as replicate sensitivity), we observed striking differences between the cell lines (Fig 10). For MCF-7, replicate sensitivity was quite similar between replicates both for individual sites (∼0.56) and clusters (∼0.79). In contrast, larger differences were observed for the other two cell lines, in particular for the A549 cell line. Here, replicate sensitivity was 0.77 and 0.93 for individual sites and clusters, respectively, for replicate 1, but only 0.29 and 0.38 for replicate 2. This means that although only a small number of poly(A) site clusters were determined for A549 in replicate 1, almost all of them were also determined in the second replicate. In all cases, replicate sensitivity was between 17% and 24% higher when looking at poly(A) site clusters instead of individual predicted sites. Finally, when comparing read counts for individual poly(A) sites identified in both replicates, we found that these were highly correlated for the MCF-7 and A549 cell line with correlation coefficients between 0.8 (for H1-hESC) and ∼0.935 (for A549 and MCF-7). However, while the actual read counts were similar between replicates for MCF-7 and H1-hESC (average fold-change 1.07), for A549 read counts in replicate 1 were smaller than in replicate 2 by a factor of ∼2.4.

**Fig 11:**
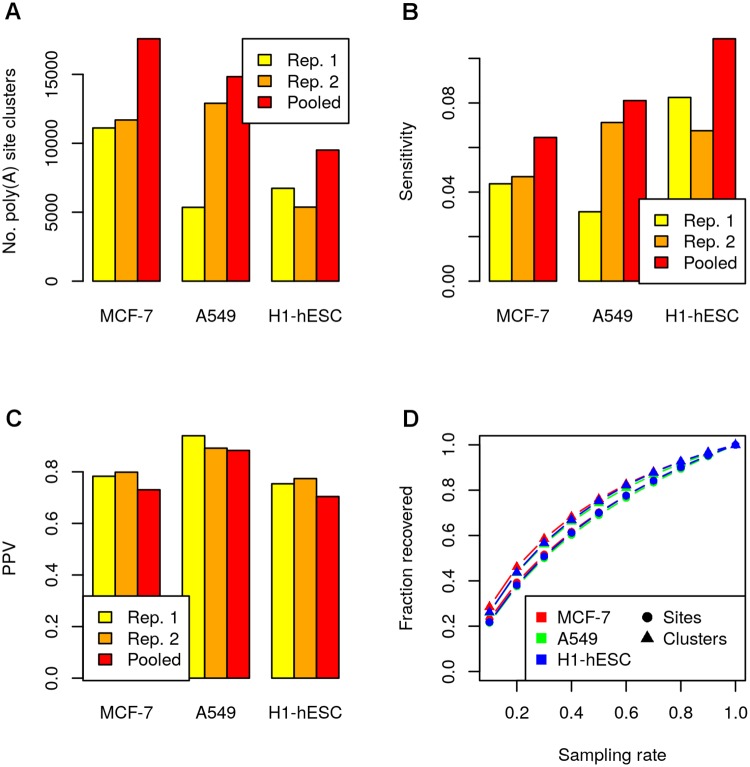
Influence of sequencing depth. (A) Number of identified poly(A) site clusters for individual replicates and the pooled sequencing data sets. Sensitivity (B) and PPV (C) for corresponding poly(A) site clusters compared to the RNA-PET gold standard. (D) Saturation in poly(A) site discovery was investigated by sampling poly(A) reads from poly(A) sites identified on the pooled data sets. The x-axis indicates which fraction of reads were sampled (sampling rate) and the y-axis shows the average fraction of the original poly(A) sites and poly(A) site clusters that were recovered across 100 repeats of sampling with the same sampling rate, respectively.

As a second analysis, we investigated how increasing sequencing depth can improve performance of poly(A) site prediction. For this purpose, we pooled both replicates for each cell line, mapped the pooled data with ContextMap 2 to identify poly(A) sites and evaluated PPV and sensitivity for the resulting poly(A) site clusters (Fig 11A–11C). The results showed that increasing sequencing depth substantially increased the number of identified poly(A) sites. However, although sequencing depth approximately doubled for all cell lines, the number of identified poly(A) site clusters increased only by around 70%. Due to the extreme variation between replicates for the A549 cell line, both lowest (for replicate 2) and highest (for replicate 1) relative increases were observed for this cell line. Sensitivity also increased by around 61% when pooling the sequencing data (Fig 11B), which is slightly less than the increase in the number of predictions. This discrepancy appears to be due to a slight reduction in PPV by around 6% (Fig 11C) and is consistent with an increasing number of low expression and consequently low confidence poly(A) sites being picked up in the pooled data. Of note, around 16% more poly(A) sites are identified in the pooled data than if we simply combine the predictions for both replicates, i.e. these sites were below the detection limit for each individual replicate.

Finally, to estimate to what extent a further increase might increase the number of identified poly(A) sites, we performed a saturation analysis by sampling poly(A) read counts for the poly(A) sites identified on the pooled data sets in the following way. For each read supporting a site, we sampled a uniformly distributed random number between 0 and 1 and retained the read if the number was smaller than the sampling rate. Here, sampling rates between 0.1 and 0.9 (in increments of 0.1) were evaluated and sampling was performed 100 times for each value of the sampling rate. After sampling the individual reads for each individual poly(A) site, we repeated the final filtering step of ContextMap 2 using the sampled read counts for each poly(A) site. This means that we again identified sets of poly(A) sites for which the distance from the first to the last site was at most the read length and retained all sites for which the corresponding set was supported by at least *r*_*s*_ = 3 poly(A) reads. Finally, the average fraction of original poly(A) sites and poly(A) site clusters that were recovered was calculated for each sampling rate (Fig 11D). Interestingly, saturation curves were very similar for all data sets as well as both individual sites and clusters. Furthermore, they showed that although the rate of increase was no longer linear, saturation was not reached yet. Thus, by increasing sequencing depth, the number of recovered poly(A) sites will likely be further increased, but with diminishing returns.

### Mapping HSV-1 poly(A) sites

Our previous results showed that the PPV of the poly(A) site mapping approach is generally very high, whereas sensitivity mostly depends on read coverage of the corresponding transcripts ends. As mentioned in the introduction, one application in which high coverage is often observed is sequencing of the RNA of an infected host cell. As infection commonly results in massive transcription of a relatively small pathogen genome, coverage of pathogen transcripts is generally high, allowing successful identification of poly(A) sites using poly(A) reads. Recently, we applied a simple poly(A) read mapping strategy to identify poly(A) sites and quantify transcription termination in HSV-1 using sequencing data of newly transcribed RNA [27]. The main distinction to the approach included in ContextMap 2 was that we first identified any read containing a poly(A) stretch and then further reduced the number of matches (many of which were false positives) by filtering based on reproducibility between different samples and replicates.

To investigate how well the poly(A) mapping strategy implemented in ContextMap 2 performs in this scenario, we applied it to all samples of newly transcribed RNA obtained during HSV-1 infection from this study (see methods). Sensitivity and PPV were evaluated by comparing predicted poly(A) sites against annotated poly(A) signal sequences in the HSV-1 genome. A predicted site was considered correct if it was within 50 nt downstream of an annotated signal sequence. For all known genes in the HSV-1 genome, a poly(A) signal sequence was annotated. As HSV-1 contains many overlapping genes on the same strand with distinct 5’ ends but the same 3’ ends, several poly(A) signals are used by more than one gene. In the following, we will refer to poly(A) signals by the corresponding gene name (or names divided by a slash in case several genes use the same poly(A) signal).

**Fig 12:**
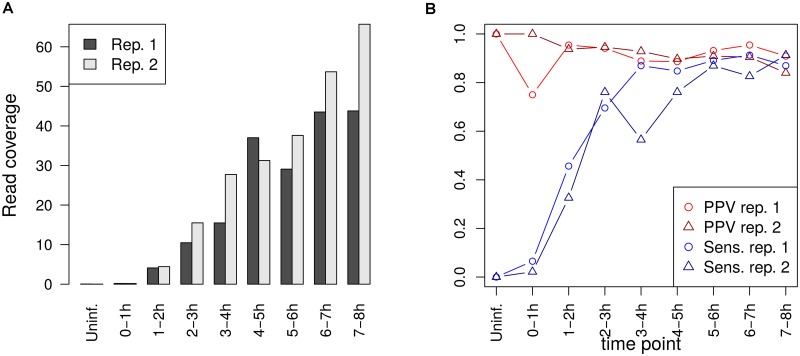
Performance of poly(A) site prediction in HSV-1. (A) Read coverage (= number of read pairs mapped divided by genome length) on the HSV-1 genome for the newly transcribed RNA samples. (B) PPV and sensitivity for poly(A) sites in HSV-1. A poly(A) site was considered a true positive if it was within 50 nt downstream of an annotated poly(A) signal and a false positive otherwise. A poly(A) signal without a predicted poly(A) site within 50 nt downstream was considered a false negative.

As can be see in Fig 12A, read coverage of the HSV-1 genome is extremely high, exceeding 10 read pairs per nucleotide already at 2-3h after infection in both replicates. Consequently, sensitivity was already much higher at this point than observed for any ENCODE data set (∼0.73) and even reached up to 0.91 for later time points (Fig 12B). In total, only two poly(A) sites were not identified in any sample, corresponding to genes *RL1* and *UL52/UL53*. In our previously published analysis, the *RL1* poly(A) site could also not be identified and the *UL52/UL53* with only few reads (at most 4). These reads were now missed, but might be recovered with less stringent parameter settings for ContextMap 2. For *RL1*, it is not surprising that no poly(A) reads could be identified as it was the least highly expressed HSV-1 genes across all time-points, with read coverage in the 1000 nt upstream of the poly(A) signal <2.7 in all samples. Other genes with low expression at the beginning of the time course, i.e. *RS1* and *LAT*, reach higher coverage late in lytic infection, thus allowing detection of the corresponding poly(A) sites. For *UL52/UL53* it was surprising that no poly(A) reads could be detected in this study (and only few in our previous analysis) as read coverage was >16 in the 1000 nt window upstream of the poly(A) signal in more than 50% of samples. This is substantially higher than for *RS1* (median read coverage 1.74 in the 1000 nt window before the poly(A) signal), whose poly(A) site was identified in both replicates as early as 1-2h after infection. It is tempting to speculate that these observation hint at differences in polyadenylation for *UL52/UL53*, but this would have to be validated experimentally.

Consistent with the results on the ENCODE data, PPV was high at all time points, exceeding 0.84 in all but one sample. In particular, in uninfected cells no poly(A) sites at all were predicted for HSV-1, despite the fact that mapping was also performed against both the human and HSV-1 genome. In total, 15 poly(A) sites were identified in the infected RNA samples that did not correspond to an annotated signal sequence. These included 2 of the 3 unannotated sites identified in our previous study, however both were only identified in only sample. Thus, they are either false positive results or only very weakly used sites. In total, only 6 poly(A) sites without annotation were identified in at least 2 samples and 2 in at least 10 samples. The latter two poly(A) sites were followed by poly(A) sequences in the genome and were located within early and relatively highly expressed genes (*UL27/UL28* and *RL2*), the combination of which likely lead to consistent false positive results across samples. We previously investigated using the presence of poly(A) sequences in the genome as a general filtering step to exclude false positive results, however found that it improved PPV only little while considerably reducing sensitivity, thus it was not further pursued. Interestingly, the predicted site within *RL2* without preceding poly(A) signal, which was both the most frequently observed false positive (14 of 16 infected samples) and the most highly expressed one (median read count 39.5 compared to 8 for the *UL27/UL28* prediction), was only slightly upstream of the known and correctly identified poly(A) site of *RL2*. Manual inspection of corresponding poly(A) reads showed that many contain poly(A) stretches longer than the poly(A) stretch in the genome, suggesting that they do actually contain part of the poly(A) read but the position of the poly(A) site was incorrectly predicted for these reads.

**Fig 13:**
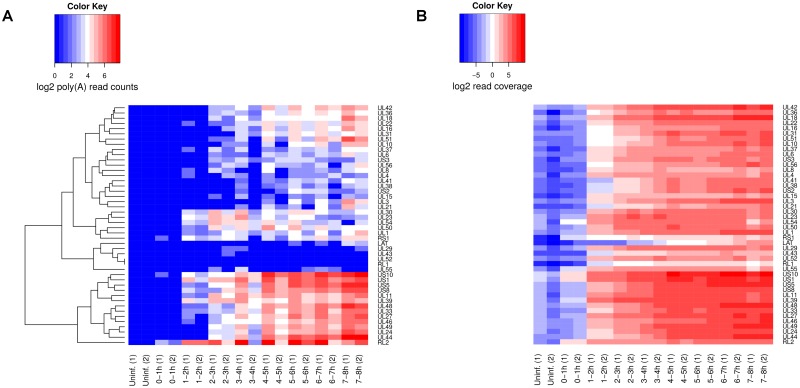
HSV-1 poly(A) site expression. (A) Heatmap of read counts (log2 scale) for predicted poly(A) sites corresponding to annotated poly(A) signals in the HSV-1 genome. Poly(A) sites are denoted by the corresponding gene name. In case a poly(A) site corresponds to more than one gene, only one gene name is given. Time points are shown on the x-axis and numbers in round brackets indicate the replicate. (B) Heatmap of read coverages (log2 scale) within 1000 nt upstream of an annotated poly(A) signal. Again, only one gene name is shown if more than one gene use the same poly(A) signal. Genes are ordered according to the clustering obtained on the read counts for HSV-1 poly(A) sites.

Finally, it should be noted that the low sensitivity at the beginning of the time course is not simply due to low coverage of the genome, but rather reflects the sequence of HSV-1 gene expression (see [45, 46]). During lytic infection, immediate-early genes are transcribed first, including *RL2* (also known as *ICP0*), *RS1* (*ICP4*), *US1* (*ICP22*), *UL54* (*ICP27*), *US12* (*ICP47*). This is followed by expression of early genes, including e.g. *UL23* (encoding for the thymidine kinase) and *UL30* (encoding the DNA polymerase), and late genes, e.g. genes encoding for structural components of the virion. Indeed, poly(A) sites of *RL2*, *RS1* and *US12* (poly(A) site shared with *US10*) were detected first, at 0-1h in one replicate and at 1-2h in both replicates (see Fig 13A, read coverage upstream of the poly(A) sites is shown in Fig 13B). At this time, *UL54* and *US1* poly(A) sites were also detected, in addition to several other poly(A) sites including those for *UL23* and *UL30*. At 2-3h, already 37 of 46 (80%) of poly(A) sites were detected in at least one replicate. The poly(A) site observed last belongs to the *LAT* gene (latency associated transcript), which is expressed during latent infection and represses lytic gene expression [47]. Both poly(A) read counts and read coverage upstream of the poly(A) site show first a low expression and then upregulation during the first 8h of lytic infection, suggesting an impending switch from lytic to latent infection.

## Conclusion

In this article, we presented an approach for mapping reads containing part of the poly(A) tail to identify poly(A) sites. Our approach essentially generalizes procedures previously applied to identify poly(A) sites for a number of herpesviruses and has been seamlessly integrated in our RNA-seq mapping software ContextMap 2.0. Thus, it allows mapping of poly(A) sites without further ado during the RNA-seq mapping process itself. We evaluated the performance of poly(A) read mapping on real-life data from the ENCODE project and compared it to a competing approach based on transcriptome assembly. This showed that depending on parameter choice, poly(A) read mapping in ContextMap 2.0 achieves considerably higher PPV and lower sensitivity compared to the competing approach or similar results, but both at significantly lower runtime.

Further analysis of predicted poly(A) sites and gold standard sites derived from RNA-PET data illustrated that sensitivity of poly(A) read mapping is likely dramatically underestimated using RNA-PET. This is evidenced by the fact that only a relatively small fraction of transcript 3’ ends identified from RNA-PET corresponds to annotated transcript 3’ ends or is downstream of canonical poly(A) signals. In contrast, poly(A) sites predicted by ContextMap 2 tend to be close to annotated transcript 3’ ends, resulting in increased sensitivity and PPV when evaluating on RNA-PET poly(A) sites that correspond to annotated transcript 3’ ends. Furthermore, frequencies of poly(A) signals upstream of predicted sites are similar to those previously reported in the literature and sensitivity improves if an alternative gold standard based on more specific sequencing of poly(A) sites is used.

Although sensitivity is likely underestimated, it remains the main bottleneck for using poly(A) read mapping to identify poly(A) sites. While our analysis suggests that sensitivity will increase with increasing sequencing depth and read length, the reduced coverage of RNA-seq on transcript ends will always provide a problem for identifying weakly expressed poly(A) sites from poly(A) reads. Even though the threshold of detection relative to gene or isoform expression will likely decrease substantially within the next years due to increased sequencing depth, this low sensitivity needs to be taken into account for any analysis of poly(A) sites based on poly(A) read mapping. Thus, while identified poly(A) sites can be considered highly reliable due to the high PPV, absence of identified poly(A) sites needs to be taken with a grain of salt, in particular if a missing poly(A) site is potentially weakly expressed.

The advantage of using poly(A) read mapping for the analysis of (alternative) polyadenylation from RNA-seq data despite this low sensitivity is that it provides direct evidence for polyadenylation in the form of poly(A) reads. This is in contrast to methods that use transcript reconstruction to determine transcript 3’ ends, such as Cufflinks [15], or methods for identifying alternative polyadenylation based on expression differences upstream of the actual poly(A) site, such as DaPars [48]. Furthermore, poly(A) site mapping can be applied to individual samples and not only to compare samples as is the case for methods based on expression differences. Moreover, since it can now be performed as part of standard read mapping, researchers may be more ready to include it in their analysis pipeline just to see what might be found. Finally, poly(A) read mapping could also be combined with Cufflinks or DaPars to provide additional evidence for transcript 3’ ends identified by these approaches or to use these alternative approaches to identify transcript 3’ ends for which no poly(A) reads can be found.

We exemplified the usefulness of poly(A) read mapping by re-analyzing a time-course of newly transcribed RNA during the first 8 hours of HSV-1 lytic expression. In this high coverage scenario, poly(A) sites were identified for almost all annotated poly(A) signals and corresponding transcript ends, resulting in a sensitivity of up to 90% at late stages of infection. Here, the time-point at which individual poly(A) sites were first detected correlated to the well-known cascade of HSV-1 gene expression. Thus, the seemingly low sensitivity at the beginning of the time-course was due to the fact that corresponding genes were not yet expressed. This example illustrates that poly(A) read mapping is already useful now for analysis of pathogen expression and transcription termination. Thus, by integrating it into our ContextMap 2 software, which already supports parallel mapping against both the host and pathogen genomes, we further extended its value for pathogen-host transcriptomics.

## Supporting Information

S1 Fig Presence of poly(A) signal sequences upstream of individual poly(A) sites. Frequency of poly(A) signal sequences were determined within a 50 nt window upstream of all identified poly(A) sites. In this case, predicted and “gold standard” poly(A) were not clustered. Results for clustered poly(A) sites are shown in Fig 9. From left to right: predictions of ContextMap 2 (all samples and replicates), predictions of KLEAT (all samples and replicates) and “gold standard” poly(A) sites identified from RNA-PET data (all samples, replicate 1) with at least 3 and 10 reads, respectively. Poly(A) signal sequences were determined as described in Fig 9.
(TIFF)

S2 Fig Illustration of mapped reads for the RNA-PET and SAPAS data for an example gene. Mapped read counts (in log scale) using ContextMap 2 are shown both for the replicate 1 of the RNA-PET and SAPAS data for MCF-7 for the GAPDH gene. Ranges of read counts are indicated in square brackets.
(TIFF)

S1 Table Comparison of PPV and sensitivity using the orginal ENCODE mapping for the RNA-PET data. (PDF)

S2 Table Evaluation results on SAPAS MCF-7 data using ContextMap 2 for mapping the gold standard. (PDF)

S3 Table Comparison of PPV and sensitivity using the BWA mapping for the RNA-PET data. (PDF)
