# Supplementary figures and images for: Prediction of Poly(A) Sites by Poly(A) Read Mapping
Source: PLoS One. 2017 Jan 30;12(1):e0170914. doi: 10.1371/journal.pone.0170914 (PMC5279776; doi:10.1371/journal.pone.0170914)

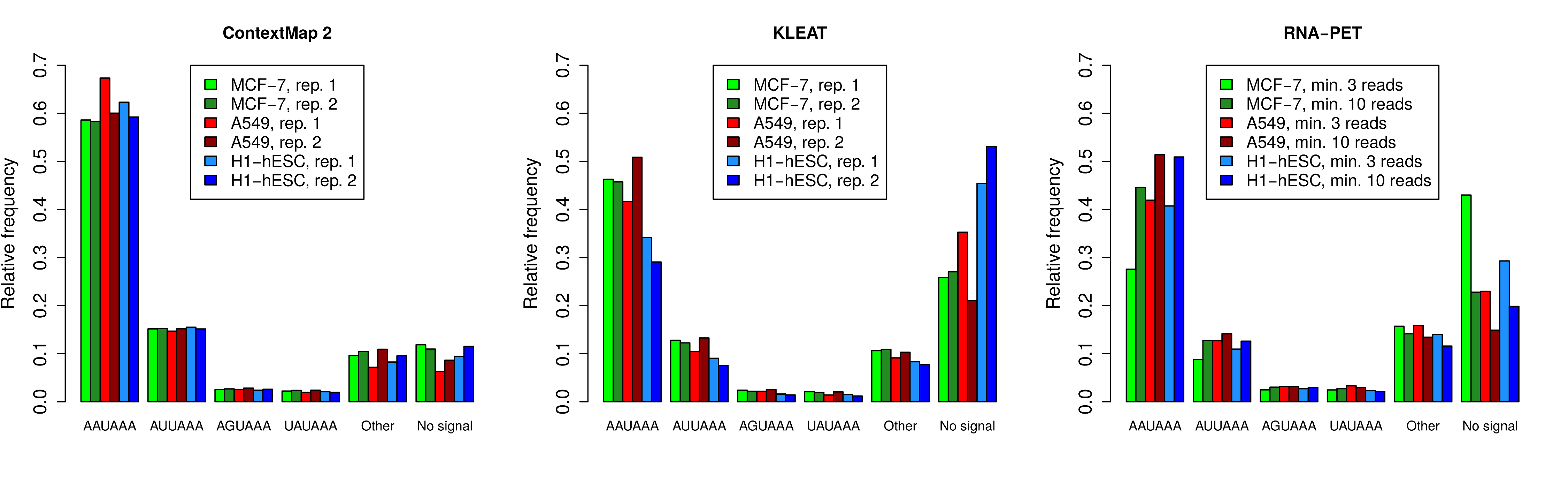

Supplement: S1 Fig — Frequency of poly(A) signal sequences were determined within a 50 nt window upstream of all identified poly(A) sites. In this case, predicted and “gold standard” poly(A) were not clustered. Results for clustered poly(A) sites are shown in Fig 9. From left to right: predictions of ContextMap 2 (all samples and replicates), predictions of KLEAT (all samples and replicates) and “gold standard” poly(A) sites identified from RNA-PET data (all samples, replicate 1) with at least 3 and 10 reads, respectively. Poly(A) signal sequences were determined as described in Fig 9. (TIFF) [file pone.0170914.s001.tiff]

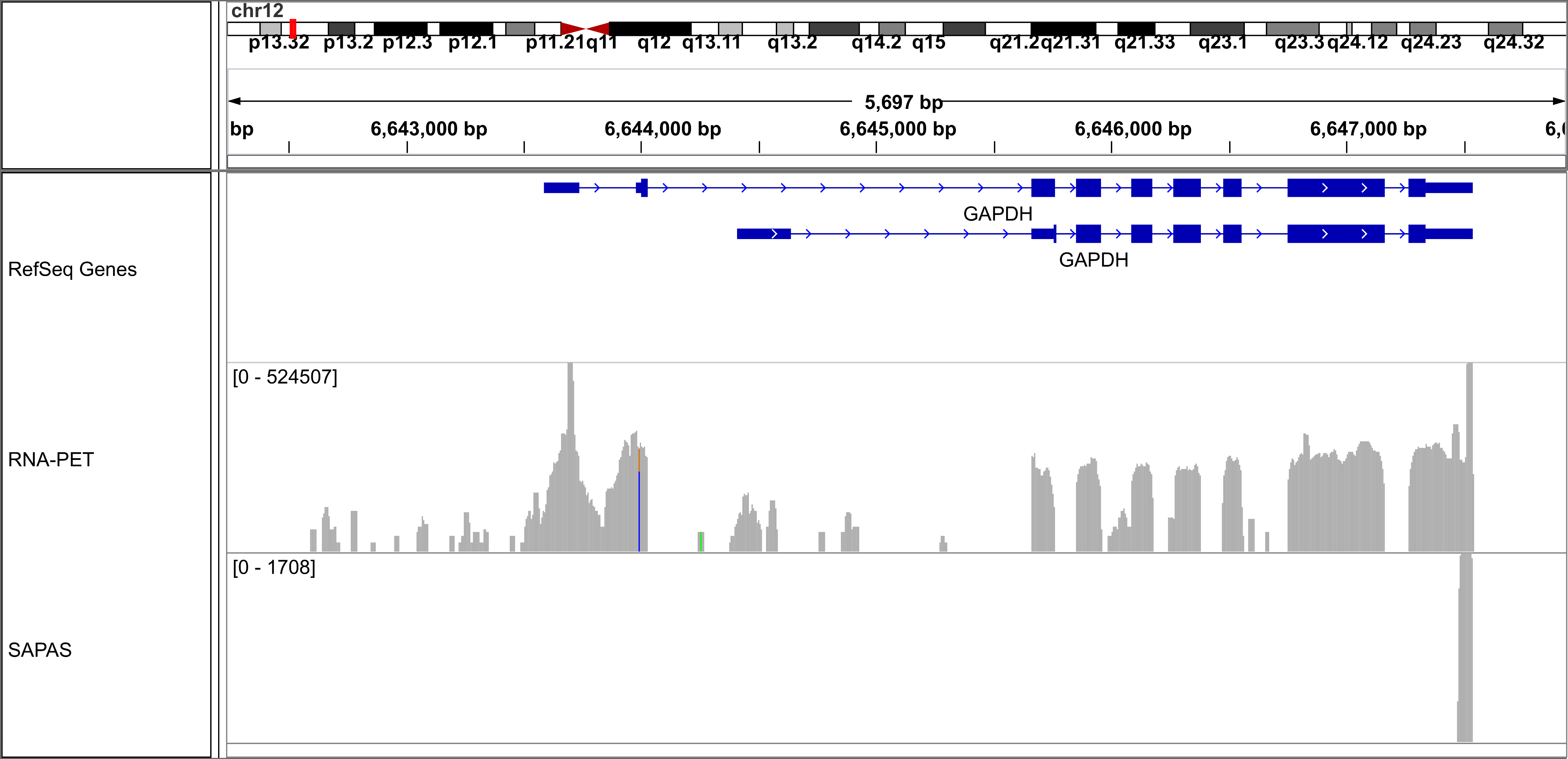

Supplement: S2 Fig — Mapped read counts (in log scale) using ContextMap 2 are shown both for the replicate 1 of the RNA-PET and SAPAS data for MCF-7 for the GAPDH gene. Ranges of read counts are indicated in square brackets. (TIFF) [file pone.0170914.s002.tiff]
