# Supplementary material for: Prediction of Poly(A) Sites by Poly(A) Read Mapping
Source: PLoS One. 2017 Jan 30;12(1):e0170914. doi: 10.1371/journal.pone.0170914 (PMC5279776; doi:10.1371/journal.pone.0170914)
Supplement: S1 Table — (PDF) [file pone.0170914.s003.pdf]

Table S1

This table shows PPV and sensitivity determined from the RNA-PET gold standard sets obtained either by mapping the data with ContextMap 2 (abbreviated as CM) or by using the original ENCODE mapping (abbreviated as ENC). In addition, differences in PPV and sensitivity are indicated. Positive values indicate that PPV or sensitivity are higher if the ContextMap 2 RNA-PET mapping is used to determine the gold standard set instead of the ENCODE RNA-PET mapping and negative values indicate that they decrease.

**Data set**	**Pred. method**	**RNA-seq rep.**	**RNA-PET rep.**	**PPV (CM)**	**PPV (ENC)**	**Diff.**	**Sens. (CM)**	**Sens. (ENC)**	**Diff.**
MCF-7									
	ContextMap	1	1	0.764	0.71	0.054	0.043	0.046	-0.002
	ContextMap	1	2	0.802	0.748	0.053	0.044	0.046	-0.002
	ContextMap	2	1	0.78	0.725	0.054	0.047	0.049	-0.003
	ContextMap	2	2	0.818	0.764	0.054	0.047	0.05	-0.003
	KLEAT	1	1	0.684	0.617	0.067	0.055	0.056	-0.001
	KLEAT	1	2	0.711	0.648	0.063	0.055	0.057	-0.002
	KLEAT	2	1	0.654	0.589	0.065	0.058	0.06	-0.001
	KLEAT	2	2	0.68	0.619	0.061	0.058	0.06	-0.002
A549									
	ContextMap	1	1	0.943	0.908	0.036	0.032	0.032	0
	ContextMap	1	2	0.936	0.9	0.037	0.03	0.033	-0.002
	ContextMap	2	1	0.896	0.852	0.044	0.073	0.073	0
	ContextMap	2	2	0.887	0.839	0.048	0.069	0.073	-0.004
	KLEAT	1	1	0.745	0.678	0.067	0.052	0.05	0.002
	KLEAT	1	2	0.74	0.67	0.07	0.049	0.05	-0.001
	KLEAT	2	1	0.815	0.753	0.062	0.08	0.077	0.002
	KLEAT	2	2	0.806	0.742	0.064	0.076	0.078	-0.002
H1-hESC									
	ContextMap	1	1	0.754	0.777	-0.024	0.082	0.027	0.056
	ContextMap	2	1	0.774	0.792	-0.018	0.068	0.022	0.046
	KLEAT	1	1	0.453	0.486	-0.032	0.114	0.038	0.076
	KLEAT	2	1	0.395	0.429	-0.034	0.094	0.032	0.062
