# Supplementary material for: Prediction of Poly(A) Sites by Poly(A) Read Mapping
Source: PLoS One. 2017 Jan 30;12(1):e0170914. doi: 10.1371/journal.pone.0170914 (PMC5279776; doi:10.1371/journal.pone.0170914)
Supplement: S2 Table — (PDF) [file pone.0170914.s004.pdf]

Table S2

PPV and sensitivity for poly(A) site predictions by ContextMap 2 and KLEAT for the MCF-7 RNA-seq data using gold standard sets obtained from the SAPAS data on MCF-7 from the study of Fu et al. For this table, mapping of the SAPAS data was performed using ContextMap 2.

**Method**	**Rep.**	**# Preds.**	**PPV**	**Sens.**
ContextMap 2	1	11,114	0.236	0.128
ContextMap 2	2	11,690	0.247	0.140
KLEAT	1	15,671	0.167	0.128
KLEAT	2	17,353	0.158	0.133
