# Supplementary material for: Prediction of Poly(A) Sites by Poly(A) Read Mapping
Source: PLoS One. 2017 Jan 30;12(1):e0170914. doi: 10.1371/journal.pone.0170914 (PMC5279776; doi:10.1371/journal.pone.0170914)
Supplement: S3 Table — (PDF) [file pone.0170914.s005.pdf]

Table S3

This table shows PPV and sensitivity determined from the RNA-PET gold standard sets obtained either by mapping the data with ContextMap 2 (abbreviated as CM) or BWA. In addition, differences in PPV and sensitivity are indicated. Positive values indicate that PPV or sensitivity are higher if the ContextMap 2 RNA-PET mapping is used to determine the gold standard set instead of the BWA RNA-PET mapping and negative values indicate that they decrease.

**Data set**	**Pred. method**	**RNA-seq rep.**	**RNA-PET rep.**	**PPV (CM)**	**PPV (BWA)**	**Diff.**	**Sens. (CM)**	**Sens. (BWA)**	**Diff.**
MCF-7									
	ContextMap	1	1	0.764	0.637	0.127	0.043	0.043	0
	ContextMap	1	2	0.802	0.69	0.111	0.044	0.045	-0.001
	ContextMap	2	1	0.78	0.656	0.124	0.047	0.047	0
	ContextMap	2	2	0.818	0.706	0.111	0.047	0.049	-0.001
	KLEAT	1	1	0.684	0.554	0.13	0.055	0.053	0.002
	KLEAT	1	2	0.711	0.595	0.116	0.055	0.055	0
	KLEAT	2	1	0.654	0.527	0.128	0.058	0.056	0.003
	KLEAT	2	2	0.68	0.562	0.118	0.058	0.057	0.001
A549									
	ContextMap	1	1	0.943	0.852	0.091	0.032	0.041	-0.009
	ContextMap	1	2	0.936	0.834	0.102	0.03	0.038	-0.007
	ContextMap	2	1	0.896	0.73	0.166	0.073	0.084	-0.011
	ContextMap	2	2	0.887	0.709	0.178	0.069	0.077	-0.008
	KLEAT	1	1	0.745	0.642	0.102	0.052	0.063	-0.011
	KLEAT	1	2	0.74	0.629	0.111	0.049	0.058	-0.009
	KLEAT	2	1	0.815	0.661	0.154	0.08	0.091	-0.011
	KLEAT	2	2	0.806	0.645	0.161	0.076	0.084	-0.009
H1-hESC									
	ContextMap	1	1	0.754	0.59	0.164	0.082	0.057	0.026
	ContextMap	2	1	0.774	0.634	0.14	0.068	0.049	0.019
	KLEAT	1	1	0.453	0.357	0.096	0.114	0.079	0.035
	KLEAT	2	1	0.395	0.33	0.065	0.094	0.069	0.025
